# Factors associated with interest in novel interfaces for upper limb prosthesis control

**DOI:** 10.1371/journal.pone.0182482

**Published:** 2017-08-02

**Authors:** Susannah M. Engdahl, Cynthia A. Chestek, Brian Kelly, Alicia Davis, Deanna H. Gates

**Affiliations:** 1 Department of Biomedical Engineering, University of Michigan, Ann Arbor, Michigan, United States of America; 2 Neurosciences Program, University of Michigan, Ann Arbor, Michigan, United States of America; 3 Department of Electrical Engineering and Computer Science, University of Michigan, Ann Arbor, Michigan, United States of America; 4 Department of Physical Medicine and Rehabilitation, University of Michigan, Ann Arbor, Michigan, United States of America; 5 University of Michigan Orthotics and Prosthetics Center, Ann Arbor, Michigan, United States of America; 6 School of Kinesiology, University of Michigan, Ann Arbor, Michigan, United States of America; University of Chicago, UNITED STATES

## Abstract

**Background:**

Surgically invasive interfaces for upper limb prosthesis control may allow users to operate advanced, multi-articulated devices. Given the potential medical risks of these invasive interfaces, it is important to understand what factors influence an individual’s decision to try one.

**Methods:**

We conducted an anonymous online survey of individuals with upper limb loss. A total of 232 participants provided personal information (such as age, amputation level, etc.) and rated how likely they would be to try noninvasive (myoelectric) and invasive (targeted muscle reinnervation, peripheral nerve interfaces, cortical interfaces) interfaces for prosthesis control. Bivariate relationships between interest in each interface and 16 personal descriptors were examined. Significant variables from the bivariate analyses were then entered into multiple logistic regression models to predict interest in each interface.

**Results:**

While many of the bivariate relationships were significant, only a few variables remained significant in the regression models. The regression models showed that participants were more likely to be interested in all interfaces if they had unilateral limb loss (p ≤ 0.001, odds ratio ≥ 2.799). Participants were more likely to be interested in the three invasive interfaces if they were younger (p < 0.001, odds ratio ≤ 0.959) and had acquired limb loss (p ≤ 0.012, odds ratio ≥ 3.287). Participants who used a myoelectric device were more likely to be interested in myoelectric control than those who did not (p = 0.003, odds ratio = 24.958).

**Conclusions:**

Novel prosthesis control interfaces may be accepted most readily by individuals who are young, have unilateral limb loss, and/or have acquired limb loss However, this analysis did not include all possible factors that may have influenced participant’s opinions on the interfaces, so additional exploration is warranted.

## Introduction

Despite the significant functional limitations that upper limb loss can impose, many individuals with upper limb loss choose not to use a prosthesis. The average prosthesis rejection rates reported in the literature are 26% for body-powered and 23% for myoelectric prostheses, although some estimates range upward of 50% [1]. Among many other concerns, individuals with upper limb loss have reported a desire for prostheses with improved dexterity (including independent movement of the fingers and arm joints, increased range of motion, and wider variety of grasp patterns) [2, 3]. The utility of such a prosthesis would be significant in comparison to most current commercially available prostheses, which permit only one degree of freedom (open/close) [4, 5] and can be cumbersome to use. Ultimately, this suggests that acceptance of a prosthesis may be improved if individuals with upper limb loss could be given multi-articulated prostheses that mimic the anatomic and physiologic complexity of the natural human arm. In fact, one survey reported that 68% of individuals who did not use a prosthesis were willing to reconsider using a prosthesis if improvements were made at a reasonable cost [3].

However, controlling a prosthesis with multiple degrees of freedom poses a significant technical challenge because it requires the collection of multiple independent control signals [6]. The development of more advanced methods for prosthesis control is an active and rapidly-advancing area of research in which many options have been proposed. Here, we present an overview of the four primary categories of these methods: myoelectric control, targeted muscle reinnervation, peripheral nerve interfaces, and cortical interfaces. (A more detailed discussion may be found in [7] or [8]). *Myoelectric control* refers to the use of electromyographic signals recorded from the skin surface over muscles in the residual limb. This method commonly relies on a “direct” control scheme in which signals from an agonist/antagonist pair of muscles are used to control a single degree of freedom in the prosthesis [9]. It is generally possible to record only two independent signals from the residual limb [4, 7] due to muscle cross-talk and co-activation, which limits the number of degrees of freedom that can be controlled. These sites may also be physiologically unrelated to the desired movement of the prosthesis [9], making the prosthesis unintuitive to use. Mode-switching (e.g., through co-contraction of the muscle pair) is one way to increase the number of degrees of freedom that can be controlled from the same recording sites [9]. A variety of other myoelectric control strategies have been proposed to avoid direct control [10], including muscle pattern recognition algorithms in which specific signal features are extracted and used to control different degrees of freedom in the prosthesis [11–13].

Some success has been documented with *targeted muscle reinnervation*, which involves surgical relocation of peripheral nerves to residual muscles (such as the pectoralis major) in order to create additional surface recording sites for myoelectric control [14, 15]. This ability to record a greater number of independent signals facilitates the use of more fully articulated prostheses than would be possible without surgical intervention. However, because the entire nerve is used to reinnervate a muscle, the number of new recording sites that can be created is limited. Furthermore, some of the original functions of the nerve may not be achievable with the reinnervated muscle [16]. As with traditional myoelectric control, pattern recognition algorithms may be used in conjunction with targeted muscle reinnervation to control prostheses with multiple degrees of freedom [14].

Some of the shortcomings of myoelectric control and targeted muscle reinnervation may be addressed by interfacing more directly with the nervous system. One approach involves the use of *peripheral nerve interfaces*, where electrodes are implanted in the residual limb to record neural signals from the peripheral nervous system. These electrodes can be placed around the nerve [17] or within the nerve [18]. The other approach uses *cortical interfaces* for which electrodes are placed on [19] or within [20] the motor cortex to record from the central nervous system. Because these approaches record from the nervous system rather than from the muscle, they may offer a higher degree of specificity [8] and can be used to collect a high volume of independent control signals.

Despite the purported advantages of targeted muscle reinnervation, peripheral nerve interfaces, and cortical interfaces, these three approaches have increased medical risk due to their surgically invasive nature. It is important to know whether individuals with upper limb loss feel that the potential advantages of having a more advanced prosthesis would outweigh the potential medical risks associated with the control interface. We recently conducted a survey of 104 individuals with upper limb loss to evaluate the interest of these individuals in noninvasive (myoelectric) and invasive (targeted muscle reinnervation, peripheral nerve interfaces, cortical interfaces) prosthesis control interfaces [21]. Most participants (83%) expressed interest in non-invasive myoelectric control. Although the invasive interfaces were comparatively less popular, many participants (≥ 39%) still expressed interest in these technologies.

Each participant’s views on the control interfaces was likely influenced by many factors. Previous literature on factors related to prosthesis acceptance lends support to this idea. The decision to use a prosthesis is thought to be motivated by a combination of predisposing characteristics, enabling resources, and established need [22, 23]. This includes a wide range of social (e.g., family support), clinical (e.g., time of fitting, training) and individual (e.g., gender, cause of limb loss) factors [1]. Given the interrelated nature of these factors, it has been difficult to develop a substantive model to describe these relationships. In fact, a review of 89 articles on factors related to prosthesis use found that there was sufficient evidence to assume a relationship between only a few factors (level of limb loss, age, and lifestyle) and prosthesis acceptance [22].

The decision to use an invasive interface for prosthesis control may be similarly complex, if not more so given the additional considerations regarding medical risk. Some participants in our previous study used the free-form comment section to describe aspects of their decision-making process (provided as supplementary material in [21]), but meaningful conclusions cannot be drawn from these comments alone. Participants may have been influenced by more factors than they could succinctly describe, or there may have been factors that influenced them without their explicit awareness (such as gender). A more systematic exploration is needed to delineate these potential relationships.

Therefore, the purpose of this work was to explore the factors associated with an individual’s interest in novel interfaces for prosthesis control. This information may help guide the development of future prostheses to specifically benefit those individuals who are most likely to accept the technology. Additionally, we investigated whether offering prosthesis functions customized to an individual’s interests could increase their willingness to try a surgical procedure for prosthesis control.

## Materials and methods

### Ethics statement

All subjects consented to participate in this study, which was granted exempt status and approved by the Institutional Review Board at the University of Michigan Medical School (HUM00077105).

### Survey development

This study used an anonymous online survey (described in [21]; full survey available in S1 Appendix) that was administered through Qualtrics (Provo, UT). The survey was initially developed based on one author’s (CC) previous experience in surveying individuals with paralysis regarding brain-machine interfaces [24]. All authors contributed to subsequent development of the survey. Descriptions of the prosthesis interfaces were written in collaboration with several other local clinicians and researchers. (It is important to note that the descriptions were simply intended to summarize the basic idea behind each interface because exact technical details continue to change as research progresses. As such, the descriptions also included a caveat about the availability of the technology.)

An initial draft of the survey was piloted on seven individuals during their appointments at the University of Michigan Orthotics and Prosthetics Center. Participants completed the survey at their own pace using a tablet computer and were allowed to provide verbal feedback on any question they did not understand while taking the survey. After completing the survey, they discussed their understanding of the questions with a researcher (SE). Most feedback reflected confusion about medical or scientific terminology used in the questions, which prompted us to simplify the language as needed (e.g., changing “trauma” to “injury”, “transhumeral” to “above elbow”, etc.).

### Survey distribution

All individuals over age 18 with upper limb loss above partial hand level were eligible to participate. The survey was advertised through various online forums and mailing lists, paper flyers at the University of Michigan Orthotics and Prosthetics Center, and the Amputee Coalition’s *inMotion* magazine. Flyers were also given to clinicians (prosthetists, physical therapists, occupational therapists) for distribution in several institutions across the United States. Finally, the survey was administered via tablet computer to patients at the University of Michigan Orthotics and Prosthetics Center.

### Survey design

The first part of the survey included questions about basic demographics, prosthesis usage, and satisfaction with functional abilities. After several early participants failed to answer all questions, the survey was updated to require a response to every presented question. However, some questions only appeared based on prior answers. For example, only participants with acquired limb loss were asked to provide their age at the time of amputation.

In the second part of the survey, participants were asked about their interest in myoelectric control (MYO), targeted muscle reinnervation (TMR), peripheral nerve interfaces (PNI), and cortical interfaces (CI). After reading a brief description of each interface, participants indicated the likelihood that they would try the interface if it offered each of six different functions. The functions were roughly ordered from basic to advanced, and the questions were phrased as: “With the procedures and risks in mind, how likely are you to have the device if it could let you <specific function>?” Responses were collected on a 5-point Likert scale from “very unlikely” to “very likely.” The six functions included: 1) moving the hand slowly, 2) rotating the wrist, 3) performing a simple grasp with the arm in any position, 4) performing multiple types of grasp in which the force could be controlled, 5) performing tasks requiring fine motor control, and 6) having touch sensation.

Although myoelectric control does not require surgical intervention, it was included as a point of contrast for the three invasive interfaces. Current myoelectric technology does not offer all of the functions that were presented in the survey, so participants were forced to respond hypothetically regarding those functions. It is possible that participants who expressed interest in trying myoelectric control to achieve more advanced functionality would still be unwilling to try an invasive interface that offered the same features.

After publication of [21], we added a question asking whether there were any additional activities that participants wanted to perform with a prosthesis. Participants who responded “yes” were asked to list the activities and rate how likely they would be to try each of the four interfaces if they could perform those tasks. The questions were phrased as: “You wrote that you think it is important that your prosthetic allows you to do the following things: <activities listed by participant>. How likely would you be to try this device if it could let you do these things?” Responses were collected on a 5-point Likert scale from “very unlikely” to “very likely.”

### Data analysis

We selected 16 factors from the survey that may have affected participants’ interest in trying the four prosthesis technologies, including 14 categorical variables (Table 1) and two continuous variables (age and time since amputation). While additional factors were available in the survey, several were excluded due to a lack of variability in the responses (i.e., ethnicity and race). The remaining factors were excluded because it was unclear how to code the responses in a way that would permit a meaningful statistical analysis. These questions allowed participants to select multiple answers (e.g., reasons for choosing not to use a prosthesis) or to provide free-form answers (e.g., current occupation), which led to considerable variability in the responses.

**Table 1:** Sample characteristics for all ordinal and nominal factors.

Factor	N (%)	Total N	Factor	N (%)	Total N
**Gender**			**Cause of Limb Loss**		
0. Male 1. Female	139 (60%) 93 (40%)	232	0. Acquired limb loss 1. Congenital limb loss	186 (80%) 46 (20%)	232
**Level of Limb Loss** ^a^			**Pain Frequency** ^d^		
1. Partial hand 2. Wrist disarticulation 3. Transradial 4. Elbow disarticulation 5. Transhumeral 6. Shoulder disarticulation 7. Forequarter	0 (0%) 20 (9%) 109 (47%) 14 (6%) 60 (26%) 13 (5%) 16 (7%)	232	1. Never 2. Less than once a month 3. Once per month 4. 2–3 times per month 5. Once per week 6. 2–3 times per week 7. Daily	50 (22%) 36 (16%) 15 (7%) 31 (13%) 10 (4%) 24 (10%) 65 (28%)	231
**Prosthesis Use**			**Side of Limb Loss** ^e^		
0. Yes 1. No	158 (68%) 74 (32%)	232	0. Nondominant arm 1. Dominant arm	76 (49%) 79 (51%)	155
**Myoelectric Use**			**Unilateral/Bilateral**		
0. Yes 1. No	71 (31%) 161 (69%)	232	0. Unilateral limb loss 1. Bilateral limb loss	197 (85%) 35 (15%)	232
**Prosthesis Type** ^b^^,^ ^c^			**Functional Satisfaction** ^f^		
1. Passive 2. Body-powered 3. Myoelectric 4. Adaptive or hybrid	21 (14%) 69 (47%) 50 (34%) 6 (4%)	146	1. Very dissatisfied 2. Dissatisfied 3. Neutral 4. Satisfied 5. Very satisfied	14 (6%) 39 (17%) 46 (20%) 99 (43%) 33 (14%)	232
**Prosthesis Satisfaction** ^c^			**Prosthesis Necessity**		
1. Very dissatisfied 2. Dissatisfied 3. Neutral 4. Satisfied 5. Very satisfied	6 (4%) 14 (10%) 28 (19%) 62 (42%) 36 (25%)	146	1. Very unnecessary 2. Unnecessary 3. Unsure 4. Necessary 5. Very necessary	32 (14%) 37 (16%) 28 (12%) 59 (25%) 76 (33%)	232
**Education**			**Lower Limb Loss**		
1. Some high school or high school degree 2. Some college or college degree 3. Post-graduate or professional degree	28 (12%) 158 (68%) 46 (20%)	232	0. Yes 1. No	35 (15%) 197 (85%)	232

List numbers indicate the coding for each factor.

^a^ refers to highest level between arms for participants with bilateral limb loss;

^b^ not determined for 12 participants who used multiple prostheses with equal frequency;

^c^ refers to most frequently used prosthesis;

^d^ refers to pain in residual limb;

^e^ not determined for participants with bilateral or congenital limb loss;

^f^ refers to overall functional ability, regardless whether prosthesis is used

The outcome measure for each interface was a dichotomous variable indicating whether or not the participant expressed interest (i.e., responded “likely” or “very likely”) to any of the six functions. Statistical analyses were performed using SPSS version 22 (IBM Corp., Armonk, NY, USA). Bivariate relationships between each factor and each outcome measure were explored using chi-squared tests (for nominal factors) and Mann-Whitney U tests (for continuous and ordinal factors). The false-discovery rate among the resulting 16 comparisons for each interface was controlled using the Benjamini-Hochberg procedure (α = 0.09).

A series of logistic regression models were created to predict each outcome measure from the set of factors. Only factors that were statistically significant in the bivariate analyses were included in the logistic regressions. Time Since Amputation, Prosthesis Type, and Prosthesis Satisfaction were not included because they were only relevant for participants with acquired limb loss or participants who used a prosthesis. Separate models that included these factors were created for the appropriate subset of participants. Participants with missing data on any factor (n = 5) were excluded from the models. Given the lack of prior investigation in this area, we chose to enter all factors into each model simultaneously (forced entry). Interaction effects were not included in this exploratory analysis.

Receiver operating characteristic (ROC) curves were used to describe each model’s ability to predict the outcome measure. ROC curves are created by plotting the true positive prediction rate against the false positive prediction rate for the model using a range of threshold parameters. The area under the ROC curve (AUC) represents the probability that the model will rank a randomly chosen positive case (i.e., a participant who expressed interest in the interface) higher than a randomly chosen negative case (i.e., a participant who did not express interest in the interface). An AUC of 0.5 indicates that the model is performing according to chance, while an AUC of 1 indicates that the model is performing perfectly.

## Results

A total of 250 individuals participated in the survey after the publication of [21]. Responses were discarded if the participant stated that they had already taken the survey (n = 14), declined participation after reading the consent form (n = 4), had only partial hand amputations (n = 8), or submitted an incomplete response (n = 75). The remaining 149 responses were combined with the 104 responses reported in [21], and all 253 responses were screened for similarities in demographic information. Twenty-one apparent duplicates were identified and removed, leaving a total sample of 232 responses.

### Interest in interfaces

Participants were considered to be interested in an interface if they stated that they would be “likely” or “very likely” to try an interface with any of the six pre-selected functions. Using this criteria, a majority of participants were interested in MYO (86%), TMR (58%), and PNI (64%), while comparatively few were interested in CI (38%). Cochran’s Q test indicated significant differences among these four percentages (p < 0.001). Post-hoc comparisons with Bonferroni corrections revealed that all pairwise combinations of percentages were significantly different except TMR and PNI (p = 0.041).

### Participant characteristics

The majority of participants were middle-aged (45 ± 15 years, N = 228), male (60%) and were educated beyond high school level (some college or college degree = 68%, post-graduate or professional degree = 20%). Most participants had unilateral (85%) and acquired (80%) limb loss, which occurred primarily at the transradial and transhumeral levels. The average time since amputation was 13 ± 14 years (N = 184). A majority of participants used a prosthesis at the time of survey completion (68%). Additional descriptive information is given in Table 1. Histograms of response frequencies for each factor are also available as supplemental material (S2 Appendix).

### Bivariate relationships

A summary of the bivariate relationships is given in Tables 2 and 3. Younger ages, lower educational achievement, decreased time since amputation, lower functional satisfaction, greater prosthesis satisfaction, higher frequency of pain, and greater perceived prosthesis necessity were all generally associated with greater interest in the interfaces. Males, participants with unilateral limb loss, participants with acquired limb loss, participants who use a prosthesis, and participants who use a myoelectric prosthesis were also more interested in the interfaces. While not every relationship was statistically significant for each interface, the direction of the significant relationships were consistent across the four interfaces.

**Table 2:** Median values of continuous and ordinal factors for interested and uninterested participants.

Factor	MYO			TMR			PNI			CI			Increased interest occurs with:
	UI	INT	p ^b^	UI	INT	p ^b^	UI	INT	p ^b^	UI	INT	p ^b^	
Age	43.0	43.0	0.189	48.5	40.0	**< 0.001**	48.5	42.0	**.001**	48.0	37.0	**< 0.001**	Decreased age
Time Since Amputation ^a^	14.5	7.5	**0.012**	12.0	7.0	**< 0.001**	14.0	7.0	**< 0.001**	11.0	6.0	**0.008**	Decreased time since amputation
Level of Limb Loss	3	3	0.843	3	3	0.951	3	3	0.678	3	3	0.782	n/a
Pain Frequency	2	4	**0.025**	2	4	**0.001**	2	4.50	**< 0.001**	3	5	**< 0.001**	Increased frequency of pain
Prosthesis Necessity	2	4	**0.005**	4	4	0.202	4	4	0.731	4	4	**0.026**	Increased perceived prosthesis necessity
Prosthesis Satisfaction ^a^	5	4	**< 0.001**	4	4	**0.008**	4	4	**0.034**	4	4	**0.037**	Decreased satisfaction
Functional Satisfaction	4	4	**0.005**	4	3	**0.006**	4	3	**0.001**	4	4	0.370	Decreased satisfaction
Education	2	2	0.419	2	2	**0.005**	2	2	0.201	2	2	**0.015**	Decreased educational attainment

UI, uninterested; INT, interested; MYO, myoelectric control; TMR, targeted muscle reinnervation; PNI, peripheral nerve interfaces; CI, cortical interfaces.

^a^ not relevant for all participants;

^b^ p values were calculated using Mann-Whitney U tests

**Table 3:** Odds ratios describing the effect of nominal factors on interest in the interfaces.

Factor	Reference Category	MYO		TMR		PNI		CI	
		Odds Ratio	p ^b^	Odds Ratio	p ^b^	Odds Ratio	p ^b^	Odds Ratio	p ^b^
Gender	Female	1.60	0.202	1.88	**0.021**	2.45	**0.001**	2.25	**0.005**
Unilateral/Bilateral	Bilateral	2.38	**0.045**	2.44	**0.015**	3.17	**0.002**	2.31	**0.046**
Cause of Limb Loss	Congenital	2.63	**0.014**	3.84	**< 0.001**	4.31	**< 0.001**	8.64	**< 0.001**
Side of Limb Loss ^a^	Nondominant	0.76	0.628	0.93	0.845	0.79	0.530	1.20	0.573
Prosthesis Use	No use	2.85	**0.004**	1.31	0.334	1.04	0.894	1.30	0.373
Prosthesis Type ^a^	n/a	n/a	0.098	n/a	0.185	n/a	0.392	n/a	0.532
Myoelectric Use	No use	18.05	**< 0.001**	0.35	0.205	1.07	0.808	1.19	0.544
Lower Limb Loss	No loss	0.64	0.332	0.62	0.190	0.49	0.103	0.71	0.390

MYO, myoelectric control; TMR, targeted muscle reinnervation; PNI, peripheral nerve interfaces; CI, cortical interfaces.

^a^ not relevant for all participants;

^b^ p values were calculated using chi-squared tests

#### Effect of quadrilateral limb loss

Exploratory analysis showed that 19 (54%) of the participants with bilateral upper limb loss were actually affected quadrilaterally (i.e., bilateral upper and bilateral lower limb loss). The remaining 16 (46%) participants with bilateral upper limb loss did not have lower limb loss. However, chi-squared tests revealed that interest in the interfaces was not significantly different between these two groups (p ≥ 0.268).

### Logistic regressions

Only a few factors proved to be significant predictors for each model (Tables 4–7). The significant predictors for MYO were Unilateral/Bilateral, Myoelectric Use, and Functional Satisfaction. The significant predictors for TMR were Age, Unilateral/Bilateral, Cause of Limb Loss, and Education. The significant predictors for PNI were Age, Gender, Unilateral/Bilateral, Cause of Limb Loss, and Functional Satisfaction. The significant predictors for CI were Age, Unilateral/Bilateral, Cause of Limb Loss, and Prosthesis Necessity.

**Table 4:** Summary of logistic regression model predicting interest in MYO.

	B ^a^	S.E.	p	Odds Ratio	95% CI for Odds Ratio	Reference Category
Unilateral/Bilateral	1.846	0.569	**0.001**	6.335	[2.08, 19.33]	Bilateral
Cause of Limb Loss	0.828	0.578	0.152	2.288	[0.74, 7.10]	Congenital
Pain Frequency	0.085	0.107	0.426	1.089	[0.88, 1.34]	n/a
Prosthesis Necessity	0.339	0.182	0.063	1.403	[0.98, 2.01]	n/a
Prosthesis Use	0.030	0.538	0.955	1.030	[0.36, 2.96]	No Use
Myoelectric Use	3.217	1.087	**0.003**	24.958	[2.96, 210.19]	No Use
Functional Satisfaction	-0.448	0.228	**0.049**	0.639	[0.41, 1.00]	n/a
(Constant)	-0.611	1.343	0.649	-	-	-

Model χ^2^ (7) = 48.3, p < 0.001.

^a^ unstandardized regression coefficient

**Table 5:** Summary of logistic regression model predicting interest in TMR.

	B ^a^	S.E.	p	Odds Ratio	95% CI for Odds Ratio	Reference Category
Age	-0.042	0.011	**0.000**	0.959	[0.94, 0.98]	n/a
Gender	0.558	0.347	0.108	1.747	[0.88, 3.45]	Female
Unilateral/Bilateral	1.106	0.427	**0.010**	3.021	[1.31, 6.98]	Bilateral
Cause of Limb Loss	1.190	0.475	**0.012**	3.287	[1.30, 8.33]	Congenital
Pain Frequency	0.030	0.074	0.690	1.030	[0.89, 1.19]	n/a
Functional Satisfaction	-0.220	0.144	0.127	0.802	[0.60, 1.06]	n/a
Education	-0.627	0.288	**0.029**	0.534	[0.30, 0.94]	n/a
(Constant)	1.977	1.067	0.064	-	-	-

Model χ^2^ (7) = 49.9, p < 0.001.

^a^ unstandardized regression coefficient

**Table 6:** Summary of logistic regression model predicting interest in PNI.

	B ^a^	S.E.	p	Odds Ratio	95% CI for Odds Ratio	Reference Category
Age	-0.045	0.012	**0.000**	0.956	[0.93, 0.98]	n/a
Gender	0.973	0.364	**0.008**	2.646	[1.30, 5.40]	Female
Unilateral/Bilateral	1.366	0.439	**0.002**	3.920	[1.66, 9.28]	Bilateral
Cause of Limb Loss	1.328	0.484	**0.006**	3.773	[1.46, 9.73]	Congenital
Pain Frequency	0.059	0.078	0.454	1.060	[0.91, 1.24]	n/a
Functional Satisfaction	-0.412	0.157	**0.008**	0.662	[0.49, 0.90]	n/a
(Constant)	1.008	0.936	0.281	-	-	-

Model χ^2^ (6) = 62.7, p < 0.001.

^a^ unstandardized regression coefficient

**Table 7:** Summary of logistic regression model predicting interest in CI.

	B ^a^	S.E.	p	Odds Ratio	95% CI for Odds Ratio	Reference Category
Age	-0.057	0.012	**0.000**	0.945	[0.92, 0.97]	n/a
Gender	0.644	0.361	0.074	1.905	[0.94, 3.86]	Female
Unilateral/Bilateral	1.029	0.487	**0.035**	2.799	[1.08, 7.27]	Bilateral
Cause of Limb Loss	2.235	0.636	**0.000**	9.346	[2.69, 32.49]	Congenital
Pain Frequency	0.009	0.077	0.909	1.009	[0.87, 1.17]	n/a
Prosthesis Necessity	0.274	0.119	**0.021**	1.316	[1.04, 1.66]	n/a
Education	-0.554	0.299	0.064	0.575	[0.32, 1.03]	n/a
(Constant)	-1.103	1.161	0.342	-	-	-

Model χ^2^ (7) = 68.2, p < 0.001.

^a^ unstandardized regression coefficient

**Fig 1:**
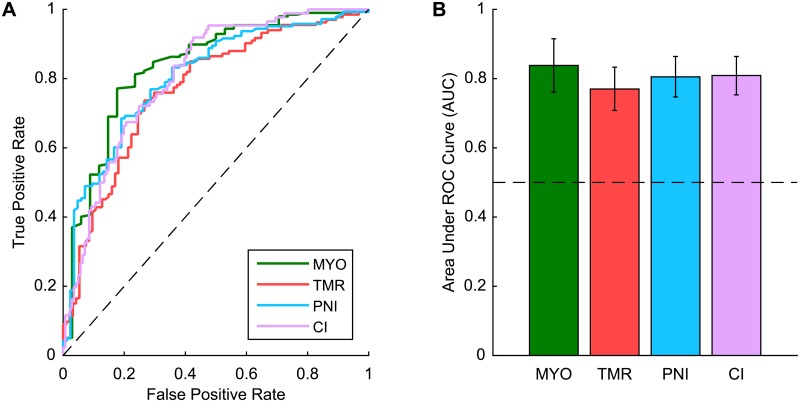
Discriminative power of the logistic regression models. (A) ROC curves for each regression model. The diagonal reference line indicates performance according to chance. (B) Area under the ROC curve for each regression model. The horizontal reference line indicates performance according to chance. Error bars represent 95% confidence intervals. (MYO = myoelectric control, TMR = targeted muscle reinnervation, PNI = peripheral nerve interfaces, CI = cortical interfaces).

All four models had good discriminatory power, as indicated by the ROC curves (Fig 1A). The AUC was similar for each model and was significantly greater than 0.5 in all cases (p < 0.001; MYO = 0.838, TMR = 0.770, PNI = 0.805, CI = 0.809) (Fig 1B).

Separate logistic regressions for participants with acquired limb loss only and for prosthesis users only are presented in the supplementary material (S3 Appendix). While the combination of significant factors varied in comparison to the models presented here, the area under the ROC curve was significantly greater (p < 0.001) than 0.5 in all cases. For the models involving participants with acquired limb loss, Time Since Amputation was a significant predictor only for MYO, TMR, and PNI. For the models involving prosthesis users, Prosthesis Satisfaction was a significant predictor only for MYO.

### Response to self-selected functions

**Fig 2:**
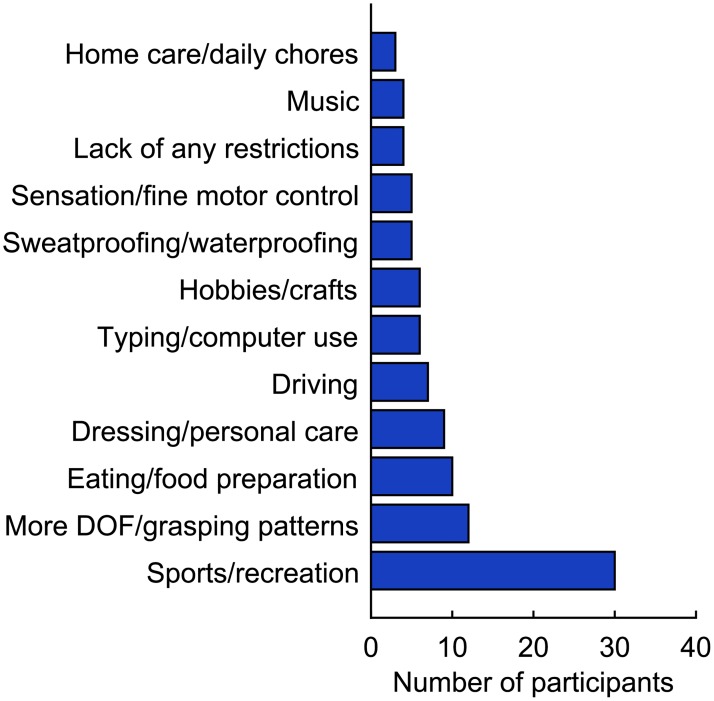
Additional categories of functions that were not already included in the survey. Participants listed additional functions that they wanted to perform with a prosthesis that were not already included in the survey. In cases where a participant mentioned multiple functions that could be classified into a single category, the participant was counted only once for that category.

**Fig 3:**
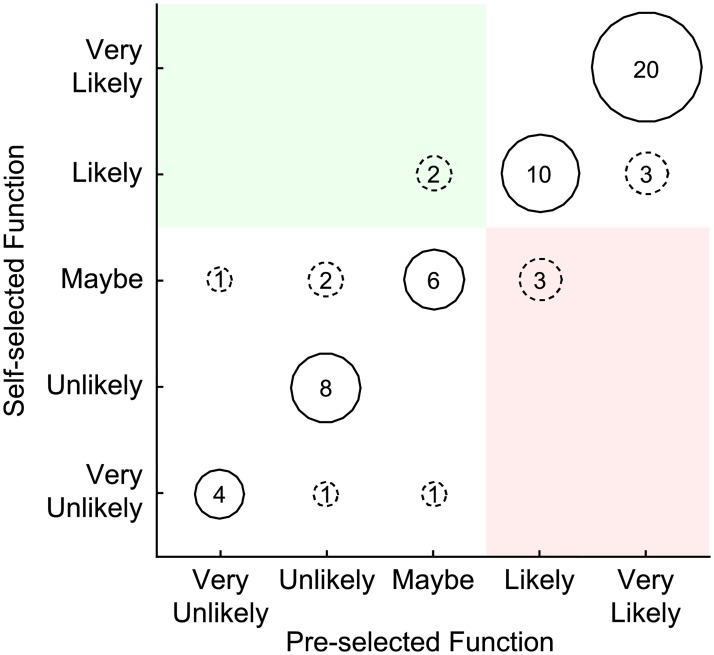
Distribution of interest in peripheral nerve interfaces depending the availability of self-selected functions. Participants indicated their interest in trying peripheral nerve interfaces if they could perform additional functions with a prosthesis that were not already included in the survey (vertical axis). These responses are presented in relation to the most interested response from the other six functions included in the survey (horizontal axis). The bubbles show the number of participants who gave each combination of responses. Dashed lines indicate a change in response. Green shading designates a change from an uninterested response (“very unlikely”, “unlikely” or “maybe”) to an interested response (“likely” or “very likely”). Red shading designates a change from an interested to uninterested response.

Only 129 participants were asked whether there were additional activities that they wanted to perform with a prosthesis, and only 61 (47%) responded affirmatively. While there was considerable diversity in the functions that were mentioned, most functions could be classified into one of 12 different categories (Fig 2; see S1 Table for a complete list). The most common functions related to sports and other recreational activities, followed by improved dexterity and grasping ability. Regardless of the interface, most participants (≥ 67%) were equally interested in the self-selected functions and pre-selected functions (representative example shown for PNI in Fig 3, see solid bubbles). Of these participants, 95% expressed interest in MYO, 47% in TMR, 63% in PNI, and 27% in CI. Few participants actually changed from uninterested to interested when the self-selected functions were added (green region in Fig 3), and some participants even changed from interested to uninterested (red region in Fig 3). However, paired t-tests revealed no significant differences in the most interested response among the pre-selected functions and responses to the self-selected functions (p ≥ 0.091).

## Discussion

The primary purpose of this work was to determine the factors associated with an individual’s interest in novel interfaces for prosthesis control. Exploratory analyses revealed several common trends, although the degree of statistical significance varied between interfaces. In general, there was greater interest among males, participants with unilateral limb loss, participants with acquired limb loss, participants who use a prosthesis, and participants who use a myoelectric prosthesis. Greater interest was also associated with younger ages, lower educational achievement, decreased time since amputation, lower satisfaction with overall functional ability, greater satisfaction with a prosthesis (if a prosthesis was used), higher frequency of pain, and greater perceived prosthesis necessity.

When these factors were used to create regression models predicting interest in each interface, many were no longer significant. In fact, each of the four regression models identified a different subset of factors that were significant predictors. The only factor that was significant for all four interfaces was Unilateral/Bilateral, where individuals with bilateral limb loss were less interested in the interfaces than those with unilateral limb loss. It might be expected that individuals with bilateral limb loss would be comparatively *more* interested given the greater severity of their impairments. However, our findings suggest these individuals may believe the associated risks (especially for the invasive interfaces) outweigh any potential benefits. Without an intact limb to rely on during recovery from surgery, training, or in case of equipment malfunction, individuals with bilateral limb loss might be particularly concerned about any loss of function. This may also explain why quadrilateral limb loss was not associated with greater interest among participants with bilateral upper limb loss.

Age and cause of limb loss were also significant predictors for all three regression models involving surgically invasive interfaces. When given the opportunity to write free-form comments about the interfaces, several participants mentioned that they would have more seriously considered the invasive interfaces if they were younger (S2 Table). These participants generally did not feel that accepting the risks associated with these interfaces would be justified at their age. The fact that age and cause of limb loss were significant may also suggest that some individuals would have been more interested in the interfaces if the technology could be implemented at the time of amputation. Some participants expressed concern about needing an additional surgery to use the interfaces, as they had already been through numerous surgeries related to their initial limb loss (S2 Table). Although the survey did not specifically ask participants to consider when they would receive the interfaces in relation to their amputation, this may have been a confounding factor.

A separate analysis explored the effect of time since amputation among participants with acquired limb loss. Generally, participants who had experienced an amputation more recently expressed greater interest in the interfaces. As individuals become more accustomed to their condition over time, they may become less interested in alternative solutions beyond what is already clinically available. Interestingly, time since amputation was a significant predictor for myoelectric control, targeted muscle reinnervation and peripheral nerve interfaces, but not cortical interfaces. This trend may suggest that opinions on cortical interfaces are particularly static over time, regardless of whether individuals have become accustomed to their amputation.

Additionally, we explored whether offering prosthesis functions customized to each participant influenced their interest in each interface. The six functions that we chose to include in the survey may not necessarily encompass everything that is considered important by individuals with upper limb loss. We hypothesized that allowing participants to identify unique functions that they valued would prompt more positive responses to the interfaces. Our findings did not support this hypothesis, as a majority of participants did not change their responses when considering their self-selected functions in comparison to the six pre-selected functions. This trend could suggest that participants believed the six pre-selected functions were already comprehensive enough to facilitate their chosen activities, or that the surgical and/or training information was more influential to their decision than the functionality. Nonetheless, it is interesting to note the diversity in the types of functions that participants chose (S1 Table).

It is important to acknowledge several limitations that may have influenced these results. First, participants’ responses to the myoelectric technology may have been biased in comparison to the other interfaces. Because myoelectric technology has been commercially available for decades, most participants were likely familiar with it already. This familiarity may have introduced additional variables into the decision-making process that were not relevant for the other, less familiar interfaces. Indeed, use of a myoelectric prosthesis was a highly significant predictor only for myoelectric control.

There were also limitations in the survey design, specifically in how the interfaces were described. We were constrained by the fact that some of the technologies do not exist outside of research labs, or do not currently exist in a form that offers all of the functions presented in this survey. It may be years before these interfaces are ready for widespread use, and the exact technical specifications are likely to change as development progresses. Consequently, we could not precisely define the training times, medical procedures, or medical risks. We also wanted the descriptions to be easily understood by individuals without a medical or scientific background, and omitted some details in order to maintain clarity. For these reasons, the descriptions were somewhat ambiguous and ultimately may have allowed participants to inaccurately infer potential benefits and risks. It would be informative to conduct another survey in the future when more technical details have been finalized so the descriptions reflect technologies that are truly available for clinical use. The results would likely differ from what we obtained here using more hypothetical descriptions.

The written comments suggest that some participants made their own inferences about the interfaces. For example, we instructed participants to assume that all the interfaces were waterproof to encourage them to evaluate each one on a broader, hypothetical level. However, waterproofing was mentioned numerous times as a desirable feature (Fig 2). Cost was also raised as a point of concern by several participants (S2 Table) even though they were instructed not to focus on cost when considering the interfaces (S1 Appendix). These findings suggest that some participants may have ignored or forgotten the instructions, or that their responses were affected by previous experiences with prosthetic technology. Cost may have been especially difficult to ignore, as many individuals with upper limb loss have difficulty obtaining adequate insurance coverage and experience a significant financial burden when acquiring, maintaining, and/or repairing their prosthesis [25]. Likewise, responses may have been influenced the participants’ prior experiences with prosthesis sockets. Socket fit is extremely important in promoting functionality and comfort [26], which many participants would have known from past prosthesis use. Although sockets were not emphasized in the survey, participants may have responded more negatively to the interfaces if they perceived that sockets were necessary and had negative opinions about traditional sockets. Concerns about sockets were in fact mentioned by several participants (S2 Table).

Similarly, the way that the functions were described may have introduced some variability in participants’ responses. Although the first 35 participants viewed a slightly different wording version (as discussed in [21]), the vast majority of participants viewed the six functions in a cumulative manner where each successive function included the previous functions. We intentionally chose this wording in order to determine whether there was a “tipping point” where participants felt that the functionality of the prosthesis would outweigh any risks. It is possible that this wording prioritized functionality in a way that participants did not necessarily agree with. Using a dichotomous outcome measure that indicated whether participants were interested in at least one of the six pre-selected functions may have reduced the impact of this wording on our analysis. Additionally, most participants did not change their response to the interfaces when considering functions customized to their interest. This suggests the six pre-selected functions already included many of the features that prosthesis users value. Nonetheless, we acknowledge that presenting the functions cumulatively may have prevented some participants from choosing the specific functions they cared about.

Finally, the sample population may not accurately represent the larger population of individuals with upper limb loss because recruitment was primarily conducted online. We expected that online recruitment would be adequate because 84% of U.S. households owned computers in 2013 and 74% used Internet in the home [27]. In an effort to include individuals who may not have computer or Internet access at home, we also made the survey available on tablet computer to patients visiting the University of Michigan Orthotics and Prosthetics Center. Our sample population matches the populations reported by other large-scale surveys in terms of several important demographic factors, including age, gender, prevalence of transradial limb loss, and prevalence of limb loss due to trauma [23, 28]. (Note that the study by Atkins et al. [28] was conducted entirely via mail). However, other characteristics of our sample population differ from what has been previously reported. Notably, the educational attainment of our participants exceeds what has been reported by Raichle et al. [29], as well as national averages reported by the U.S. Census Bureau [30]. Census records from 2015 indicate that most adults (88%) had at least a high school degree, while 33% had at least a bachelor’s degree [30]. In contrast, 99% of our participants had at least a high school degree and 53% had at least a bachelor’s degree. This may indicate a sampling bias, as computer ownership and Internet use tends to be lower in households with lower educational attainment [27]. Furthermore, computer ownership and home Internet use tends to be less common in Hispanic households compared to white, non-Hispanic households [27]. Since our sample population was predominately white and non-Hispanic, this may be further evidence of a sampling bias. The results of this work should be generalized carefully given these limitations.

## Conclusions

Our work has demonstrated that several factors are consistently associated with interest in novel interfaces for upper limb prosthesis control. Younger age, acquired limb loss, and unilateral limb loss were related to greater interest in surgically invasive interfaces. Interest in noninvasive myoelectric control was also associated with unilateral limb loss, as well as current use of a myoelectric prosthesis. Knowledge of these associations may be helpful to research efforts. For examples, researchers could try to involve individuals with these characteristics in testing and assessment of future devices. The information regarding specific benefits, medical risks, and training procedures that is gained as a result of this testing may eventually encourage those who are currently not interested to consider these interfaces.

Collectively, the work reported here and in our earlier paper [21] advances the literature in several important ways. Although it has been reported that individuals with upper limb loss are interested in novel interfaces for prosthesis control (e.g., [3]), our earlier paper was the first to actually quantify this interest. Our current analysis expands on those findings by identifying factors associated with the participants’ interest. The propensity of prosthesis developers to pursue new technologies before the end users’ needs have been clearly articulated is a detriment to individuals with limb loss [3, 31], who may reject technologies which fail to meet their demands. Our work is valuable in this context, as it helps elucidate the perspectives of individuals with upper limb loss. However, we also recommend that additional studies are done to explore patient opinions in greater detail. It is clear from our work that a single survey is insufficient to understand every factor that motivates an individual’s interest in new prosthesis technologies. A variety of other factors relating to the individual’s medical history, lifestyle, and psychosocial state should be considered.

## Supporting information

S1 Appendix Full survey. Participants were not necessarily presented with every question available in the full survey.
(PDF)

S2 Appendix Distribution plots for the factors and outcome measures. (PDF)

S3 Appendix Additional logistic regression models. (PDF)

S1 Table All self-selected functions listed by 61 participants. These responses are in their original form and have not been edited for grammar or spelling.
(XLSX)

S2 Table All written comments regarding the interfaces. Responses that were already published in [21] are not included. The comments are in their original form, although information that could potentially reveal the participant’s identity has been redacted.
(XLSX)
