# Supplementary material for: Factors associated with interest in novel interfaces for upper limb prosthesis control
Source: PLoS One. 2017 Aug 2;12(8):e0182482. doi: 10.1371/journal.pone.0182482 (PMC5540477; doi:10.1371/journal.pone.0182482)
Supplement: S1 Appendix — Participants were not necessarily presented with every question available in the full survey. (PDF) [file pone.0182482.s001.pdf]

Patient Satisfaction and Design Priorities for Upper Extremity Prosthetic Technology

The goal of this study is to determine which features are most important in a prosthetic device and to evaluate interest in new devices that are currently being developed. You are invited to participate in this research project because you have been identified as a person with upper extremity limb loss. This research project is being conducted by Dr. Deanna Gates and Dr. Cynthia Chestek at the University of Michigan.

Your participation in this research study is voluntary. You may choose not to participate. If you decide to participate in this research survey, you may withdraw at any time. If you decide not to participate in this study or if you withdraw from participating at any time, you will not be penalized.

The procedure involves filling out a survey that will take approximately 20 minutes. Your responses will be confidential and we do not collect identifying information such as your name, email address or IP address. The survey will include questions related to basic demographics, the effect of limb loss on employment, current prosthetic usage, important prosthetic characteristics, and new prosthetic technology.

To help protect your confidentiality, the survey will not contain information that will personally identify you. The results of this study will be used for scholarly purposes only and may be shared with University of Michigan representatives.

If you have any questions about the research study, please contact Dr. Gates at gatesd@umich.edu. This research has been reviewed according to the University of Michigan IRB-MED procedures for research involving human subjects.

ELECTRONIC CONSENT: Please select your choice below. Clicking on the "agree" button below indicates that:
• You have read the above information
• You voluntarily agree to participate
• You are at least 18 years of age
If you no longer wish to participate in the research study, please decline participation by clicking on the "Disagree" button.
❍ Agree
❍ Disagree

Have you taken this survey before? (If you have already taken this survey, please do not take it again.)
❍ Yes
❍ No

What is your age (in years)?

What is your gender?
- ❍ Male
- ❍ Female

Please specify your ethnicity.
- ❍ Hispanic or Latino
- ❍ Not Hispanic or Latino

Please specify your race (check all that apply).
- ❑ American Indian/Alaska Native
- ❑ Asian
- ❑ Native Hawaiian or Other Pacific Islander
- ❑ Black or African American
- ❑ White
- ❑ Other

Which is your dominant hand? (If applicable, please specify your dominant hand prior to amputation.)
- ❍ Right hand
- ❍ Left hand
- ❍ Both hands

Which arm is affected by limb loss?
- ❍ Right
- ❍ Left
- ❍ Both

What is the level of limb loss in your right arm?

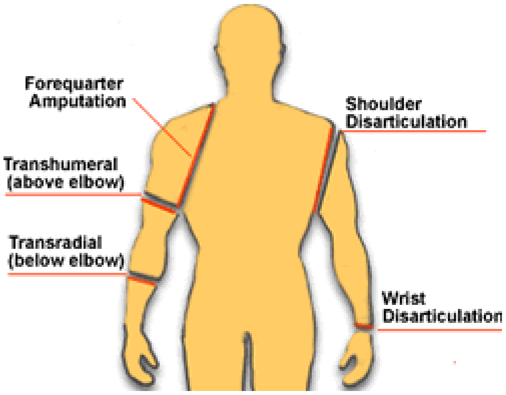

- ❍ Above the shoulder (forequarter amputation) - shoulder blade and collarbone are removed
- ❍ At the shoulder (shoulder disarticulation)
- ❍ Above elbow (transhumeral) - between the elbow and shoulder
- ❍ At the elbow (elbow disarticulation)
- ❍ Below elbow (transradial) - between the elbow and wrist
- ❍ At the wrist (wrist disarticulation)
- ❍ Partial hand - finger, thumb, or part of the hand below the wrist

What is the level of limb loss in your left arm?

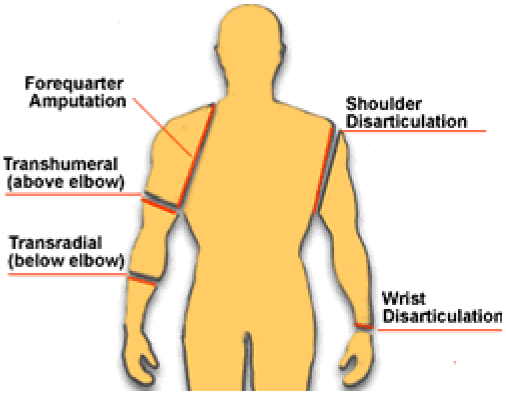

- ❍ Above the shoulder (forequarter amputation) - shoulder blade and collarbone are removed
- ❍ At the shoulder (shoulder disarticulation)
- ❍ Above elbow (transhumeral) - between the elbow and shoulder
- ❍ At the elbow (elbow disarticulation)
- ❍ Below elbow (transradial) - between the elbow and wrist
- ❍ At the wrist (wrist disarticulation)
- ❍ Partial hand - finger, thumb, or part of the hand below the wrist

What is the level of limb loss in your arm?

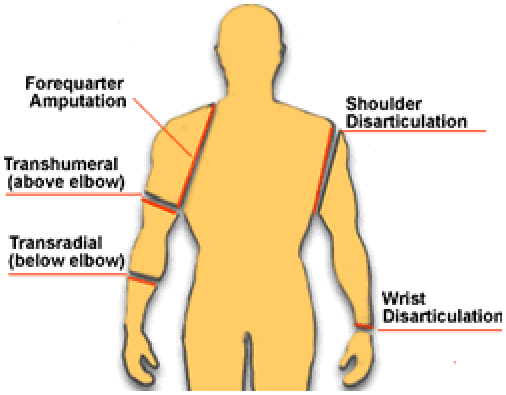

- ❍ Above the shoulder (forequarter amputation) - shoulder blade and collarbone are removed
- ❍ At the shoulder (shoulder disarticulation)
- ❍ Above elbow (transhumeral) - between the elbow and shoulder
- ❍ At the elbow (elbow disarticulation)
- ❍ Below elbow (transradial) - between the elbow and wrist
- ❍ At the wrist (wrist disarticulation)
- ❍ Partial hand - finger, thumb, or part of the hand below the wrist

What is the reason for your limb loss? (check all that apply)

- ❑ Vascular
- ❑ Cancer
- ❑ Injury
- ❑ Birth
- ❑ Infection/disease
- ❑ Other (please specify) ____________________

How old were you at the time of your amputation?

How often do you currently experience pain in your residual limb (stump)?

- ❍ Never
- ❍ Less than once a month
- ❍ Once a month
- ❍ 2-3 times a month
- ❍ Once a week
- ❍ 2-3 times a week
- ❍ Daily

Do you have a lower limb amputation?

- ❍ Yes
- ❍ No

Which leg is affected by limb loss?

- ❍ Left
- ❍ Right
- ❍ Both

What is the level of limb loss in your right leg?

- ❍ At the hip (hip disarticulation)
- ❍ Above the knee (transfemoral) - between the knee and hip
- ❍ At the knee (knee disarticulation)
- ❍ Below the knee (transtibial) - between the ankle and knee
- ❍ At the ankle (ankle disarticulation)
- ❍ Partial foot - toes or part of foot below ankle

What is the level of limb loss in your left leg?

- ❍ At the hip (hip disarticulation)
- ❍ Above the knee (transfemoral) - between the knee and hip
- ❍ At the knee (knee disarticulation)
- ❍ Below the knee (transtibial) - between the ankle and knee
- ❍ At the ankle (ankle disarticulation)
- ❍ Partial foot - toes or part of foot below ankle

What is the level of limb loss in your leg?

- ❍ At the hip (hip disarticulation)
- ❍ Above the knee (transfemoral) - between the knee and hip
- ❍ At the knee (knee disarticulation)
- ❍ Below the knee (transtibial) - between the ankle and knee
- ❍ At the ankle (ankle disarticulation)
- ❍ Partial foot - toes or part of foot below ankle

What is the highest level of education you have completed?

- ❍ Some high school
- ❍ High school diploma/GED
- ❍ Some college
- ❍ 2-year college degree (Associate)
- ❍ 4-year college degree (BA, BS)
- ❍ Master's degree
- ❍ Doctoral degree
- ❍ Professional degree (MD, JD, etc.)

What was your employment status at the time of amputation?

- ❍ Employed, full-time
- ❍ Employed, part-time
- ❍ Unemployed
- ❍ Student
- ❍ Homemaker
- ❍ Retired
- ❍ Other (please specify) ____________________

What was your occupation at the time of your amputation?

What is your current employment status?

- ❍ Employed, full-time
- ❍ Employed, part-time
- ❍ Disability leave
- ❍ Unemployed
- ❍ Homemaker
- ❍ Student
- ❍ Retired
- ❍ Other (please specify) ____________________

What is your current occupation?

Is your current unemployment related to your limb loss?

- ❍ Yes
- ❍ No
- ❍ In part

Have you ever had to switch jobs due to your limb loss?

- ❍ Yes
- ❍ No

How did limb loss affect your previous job?

How necessary is an upper limb prosthesis in your everyday life?

- ❍ Very unnecessary
- ❍ Unnecessary
- ❍ Unsure
- ❍ Necessary
- ❍ Very necessary

Do you currently use an upper limb prosthesis?
- ❍ Yes
- ❍ No

Do you use an upper limb prosthesis on both arms?
- ❍ Yes
- ❍ No

Why don't you currently use an upper limb prosthesis? (check all that apply)
- ❑ Equally as functional or more functional without a prosthetic device
- ❑ Past devices didn't improve function
- ❑ Past devices too uncomfortable
- ❑ Devices not affordable
- ❑ Appearance
- ❑ Other (please specify) ____________________

How satisfied are you with your overall ability to do the things you would like to do?
- ❍ Very Dissatisfied
- ❍ Dissatisfied
- ❍ Neutral
- ❍ Satisfied
- ❍ Very Satisfied

Which types of prosthetic devices do you currently use? (check all that apply)
- ❑ Passive: Similar in appearance to the non-affected arm and replaces what was lost. It provides simple aid in balancing and carrying.
- ❑ Body Powered/Conventional: Operated by a harness system that is controlled by specific body movements.
- ❑ Myoelectric/External Power: Powered by a battery system and is controlled by muscle activity.
- ❑ Hybrid: Combines the use of body power and external power
- ❑ Adaptive/Recreational: Customized for a specific function or recreational activity.

Which types of prosthetic devices do you currently use on your right arm? (check all that apply)
- ❑ Passive: Similar in appearance to the non-affected arm and replaces what was lost. It provides simple aid in balancing and carrying.
- ❑ Body Powered/Conventional: Operated by a harness system that is controlled by specific body movements.
- ❑ Myoelectric/External Power: Powered by a battery system and is controlled by muscle activity.
- ❑ Hybrid: Combines the use of body power and external power
- ❑ Adaptive/Recreational: Customized for a specific function or recreational activity.

Which types of prosthetic devices do you currently use on your left arm? (check all that apply)

- ❑ Passive: Similar in appearance to the non-affected arm and replaces what was lost. It provides simple aid in balancing and carrying.
- ❑ Body Powered/Conventional: Operated by a harness system that is controlled by specific body movements.
- ❑ Myoelectric/External Power: Powered by a battery system and is controlled by muscle activity.
- ❑ Hybrid: Combines the use of body power and external power
- ❑ Adaptive/Recreational: Customized for a specific function or recreational activity.

How often do you wear the passive device?

- ❍ Never
- ❍ Once in a while
- ❍ Half of the time
- ❍ A lot of the time
- ❍ Almost all of the time

How long have you used this passive device? (Please specify months or years)

Why did you choose the passive device? (check all that apply)

- ❑ Doctor recommended
- ❑ Most functional
- ❑ Comfort
- ❑ Visual appeal
- ❑ Cost
- ❑ Other (please specify) ____________________

How satisfied are you with your current passive device?

- ❍ Very Dissatisfied
- ❍ Dissatisfied
- ❍ Neutral
- ❍ Satisfied
- ❍ Very Satisfied

How often do you wear the body powered device?

❍ Never
❍ Once in a while
❍ Half of the time
❍ A lot of the time
❍ Almost all of the time

How long have you used this body powered device? (Please specify months or years)

Why did you choose the body powered device? (check all that apply)

❑ Doctor recommended
❑ Most functional
❑ Comfort
❑ Visual appeal
❑ Cost
❑ Other (please specify) ____________________

How satisfied are you with your current body powered device?

❍ Very Dissatisfied
❍ Dissatisfied
❍ Neutral
❍ Satisfied
❍ Very Satisfied

How often do you wear the myoelectric device?

- ❍ Never
- ❍ Once in a while
- ❍ Half of the time
- ❍ A lot of the time
- ❍ Almost all of the time

How long have you used this myoelectric device? (Please specify months or years)

Why did you choose the myoelectric device? (check all that apply)

- ❑ Doctor recommended
- ❑ Most functional
- ❑ Comfort
- ❑ Visual appeal
- ❑ Cost
- ❑ Other (please specify) ____________________

How satisfied are you with your current myoelectric device?

- ❍ Very Dissatisfied
- ❍ Dissatisfied
- ❍ Neutral
- ❍ Satisfied
- ❍ Very Satisfied

How often do you wear the hybrid device?

❍ Never
❍ Once in a while
❍ Half of the time
❍ A lot of the time
❍ Almost all of the time

How long have you used this hybrid device? (Please specify months or years)

Why did you choose the hybrid device? (check all that apply)

❑ Doctor recommended
❑ Most functional
❑ Comfort
❑ Visual appeal
❑ Cost
❑ Other (please specify) ____________________

How satisfied are you with your current hybrid device?

❍ Very Dissatisfied
❍ Dissatisfied
❍ Neutral
❍ Satisfied
❍ Very Satisfied

How often do you wear the recreational device?

- ❍ Never
- ❍ Once in a while
- ❍ Half of the time
- ❍ A lot of the time
- ❍ Almost all of the time

How long have you used this recreational device? (Please specify months or years)

Why did you choose the recreational device? (check all that apply)

- ❑ Doctor recommended
- ❑ Most functional
- ❑ Comfort
- ❑ Visual appeal
- ❑ Cost
- ❑ Other (please specify) ____________________

How satisfied are you with your current recreational device?

- ❍ Very Dissatisfied
- ❍ Dissatisfied
- ❍ Neutral
- ❍ Satisfied
- ❍ Very Satisfied

How satisfied are you with your overall ability to do the things you would like to do?

- ❍ Very Dissatisfied
- ❍ Dissatisfied
- ❍ Neutral
- ❍ Satisfied
- ❍ Very Satisfied

How important is it to you that your prosthetic device allows you to do the following things?

	Very unimportant	Unimportant	Neutral	Important	Very important
Open and close your hand slowly	❍	❍	❍	❍	❍
Do all the above AND rotate your wrist	❍	❍	❍	❍	❍
Do all the above AND move to any location in your workspace and perform a simple grasp	❍	❍	❍	❍	❍
Do all the above AND perform several types of grasps, in which you can control the amount of force used	❍	❍	❍	❍	❍
Do all the above AND perform tasks that require fine motor control (such as writing with a pen or typing)	❍	❍	❍	❍	❍
Do all the above AND have touch sensation in the missing limb	❍	❍	❍	❍	❍

Besides the activities listed above, are there other activities that you would like to do with your prosthetic device?

- ❍ Yes
- ❍ No

Please list these activities.

The next series of questions will ask you about whether you would be willing to try new prosthetic devices or undergo surgery if you could perform certain functions. Not all of these devices are currently available to the public.

When considering each device or procedure, please:
Focus on the capabilities of the device and risks of the procedure, rather than the potential monetary cost.
Assume the device is appropriate for your level of amputation (even if the picture does not show your level of amputation).
Assume you have no medical restrictions that would prevent you from using the device.
Assume the device is waterproof and looks similar to a real arm.

Myoelectric Device Controlled by Muscles in the Arm
Device Description: This device has sensors embedded in the socket of the prosthesis that touch your skin. The sensors detect electric signals that are produced when you contract your muscles. These electric signals are used to control movement of the prosthesis.

Medical Procedure: This device requires no surgery to use.

Training Time: About 3 months.

Potential Risks: There is no risk associated with using this device.

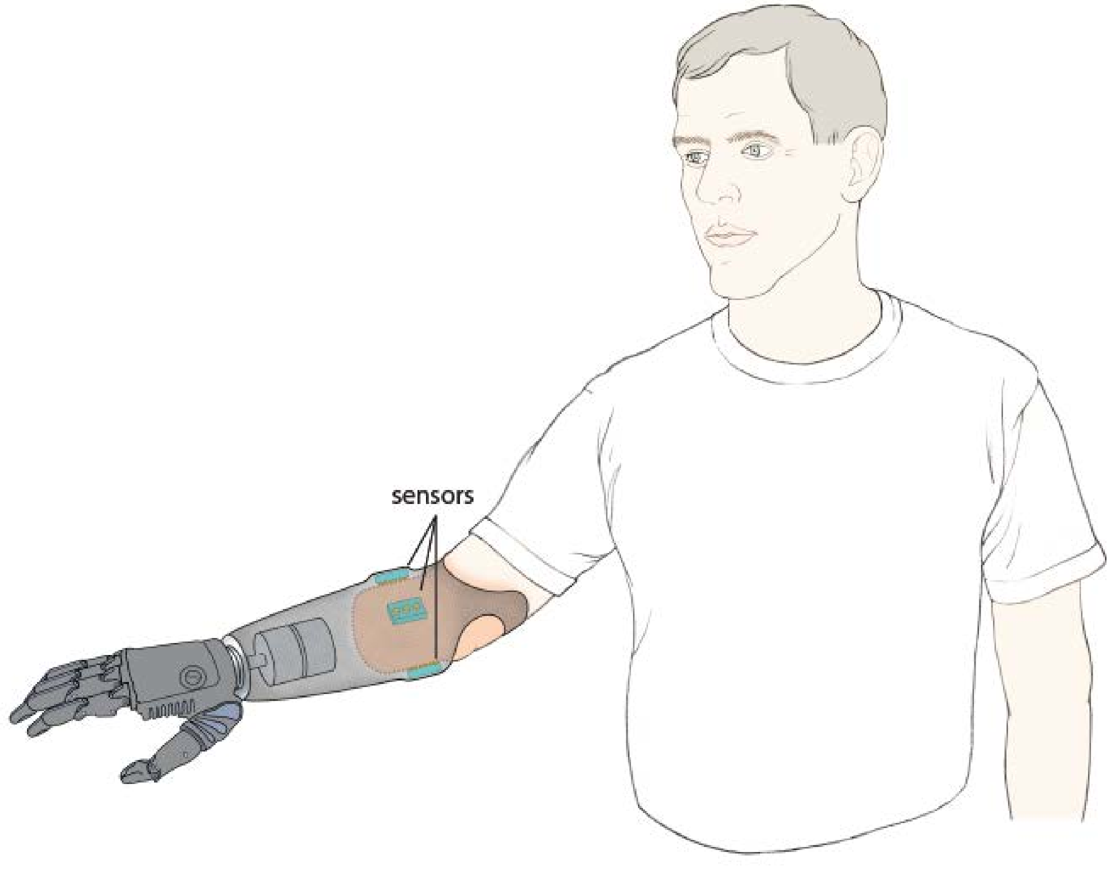

	With the procedure and risks in mind, how likely are you to have the device if it could let you:				
	Very Unlikely	Unlikely	Maybe	Likely	Very Likely
Open and close your hand slowly	❍	❍	❍	❍	❍
Do all the above AND rotate your wrist	❍	❍	❍	❍	❍
Do all the above AND move to any location in your workspace and perform a simple grasp	❍	❍	❍	❍	❍
Do all the above AND perform several types of grasps, in which you can control the amount of force used	❍	❍	❍	❍	❍
Do all the above AND perform tasks that require fine motor control (such as writing with a pen or typing)	❍	❍	❍	❍	❍
Do all the above AND have touch sensation in the missing limb	❍	❍	❍	❍	❍

You wrote that you think it is important that your prosthetic allows you to do the following things: ${q://QID101/ChoiceTextEntryValue}   How likely would you be to try this device if it could let you do these things?

- Very Unlikely
- Unlikely
- Maybe
- Likely
- Very Likely

Do you have any additional thoughts on this device? We are interested in understanding why you are interested or why you are not interested in trying this device.

Surgery that Moves Nerves into Different Muscles

Device Description: This device operates like the myoelectric device described previously, except that you will need to have a surgical procedure before you can use it. In this procedure, the nerves that originally controlled your amputated limb are moved and connected to muscles that have not been amputated. As a result, these muscles will contract if you attempt to move your missing limb. Sensors in the prosthesis detect electric signals from these contractions and these signals are used to control movement of the prosthesis.

Medical Procedure: The nerves are moved with a surgical procedure that requires making incisions in the arm. This procedure requires a hospital stay of about 1-3 days. The incision area will heal in about 2 weeks and become unnoticeable. After the surgery, you must wait 5-6 months to allow the nerves to regrow before you can begin training with the device.

Training Time: About 1 month.

Potential Risks: There is about a 2-3% risk of a minor medical problem (such as bruising) and a small risk of a major complication.

** This device is not currently available to the public.

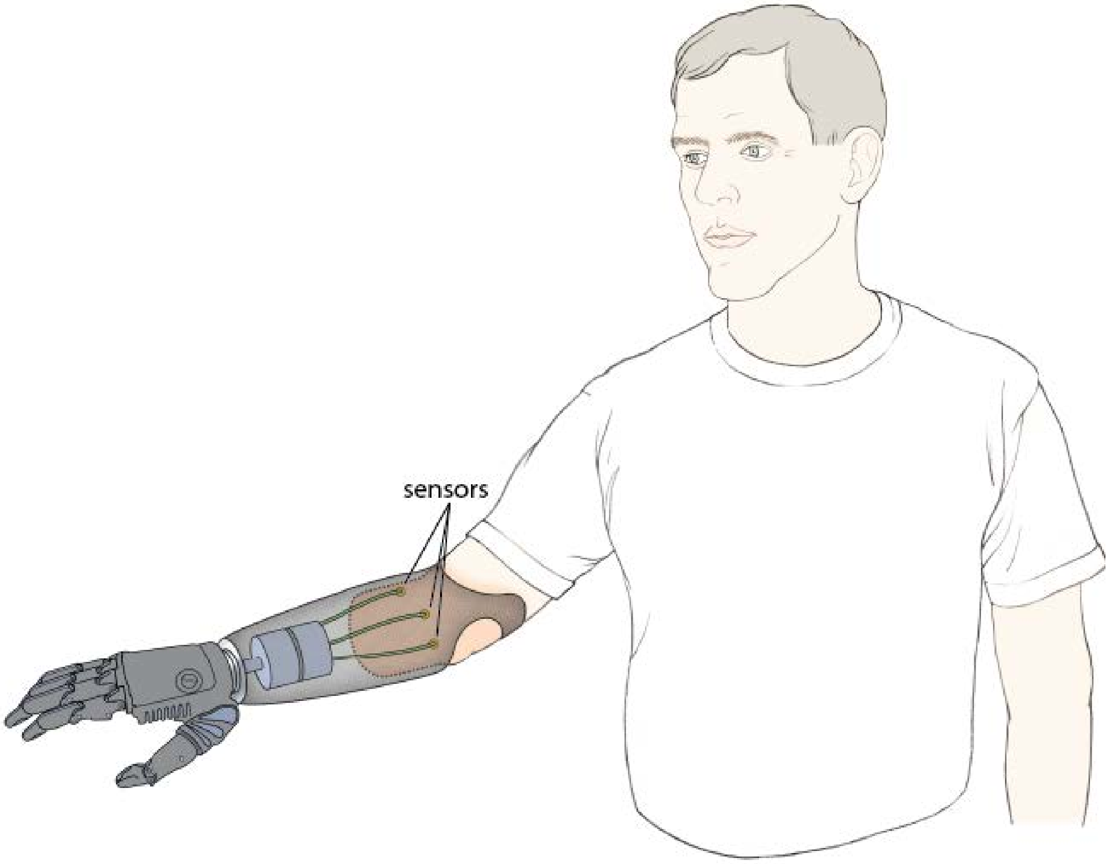

	With the procedure and risks in mind, how likely are you to have the device if it could let you:				
	Very Unlikely	Unlikely	Maybe	Likely	Very Likely
Open and close your hand slowly	❍	❍	❍	❍	❍
Do all the above AND rotate your wrist	❍	❍	❍	❍	❍
Do all the above AND move to any location in your workspace and perform a simple grasp	❍	❍	❍	❍	❍
Do all the above AND perform several types of grasps, in which you can control the amount of force used	❍	❍	❍	❍	❍
Do all the above AND perform tasks that require fine motor control (such as writing with a pen or typing)	❍	❍	❍	❍	❍
Do all the above AND have touch sensation in the missing limb	❍	❍	❍	❍	❍

You wrote that you think it is important that your prosthetic allows you to do the following things: ${q://QID101/ChoiceTextEntryValue} How likely would you be to try this device if it could let you do these things?

- Very Unlikely
- Unlikely
- Maybe
- Likely
- Very Likely

Do you have any additional thoughts on this device? We are interested in understanding why you are interested or why you are not interested in trying this device.

Wireless Device Implanted in the Arm

Device Description: This device is an array of 16 small sensors implanted in the arm. During a surgical procedure, small pieces of muscle are connected to the nerves that originally controlled your amputated limb. Sensors are then wrapped around these nerves and muscles. The sensors detect electric signals that are produced if you attempt to move your missing limb. These electric signals are used to control movement of the prosthesis.

Medical Procedure: This device is put in with a surgical procedure that requires opening incisions on the arm. This procedure requires a hospital stay of about 1 day. The incision area will heal in about 2 weeks and become unnoticeable.

Training Time: About 1 week.

Potential Risks: There is about a 2-3% risk of a minor medical problem (such as bruising or infection) and a small risk of a major complication. There is a small risk that additional surgery would be required to repair breakages in the system.

** This device is not currently available to the public.

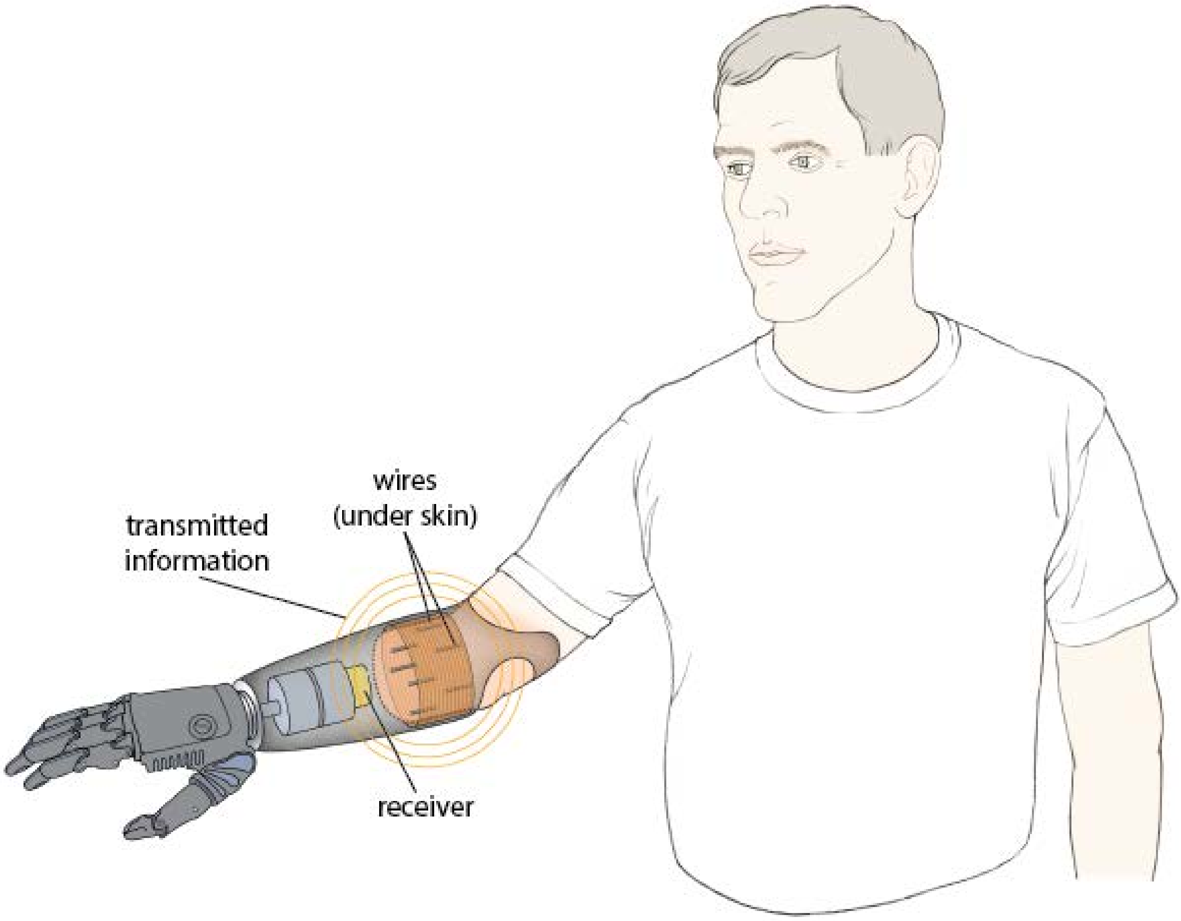

	With the procedure and risks in mind, how likely are you to have the device if it could let you:				
	Very Unlikely	Unlikely	Maybe	Likely	Very Likely
Open and close your hand slowly	❍	❍	❍	❍	❍
Do all the above AND rotate your wrist	❍	❍	❍	❍	❍
Do all the above AND move to any location in your workspace and perform a simple grasp	❍	❍	❍	❍	❍
Do all the above AND perform several types of grasps, in which you can control the amount of force used	❍	❍	❍	❍	❍
Do all the above AND perform tasks that require fine motor control (such as writing with a pen or typing)	❍	❍	❍	❍	❍
Do all the above AND have touch sensation in the missing limb	❍	❍	❍	❍	❍

You wrote that you think it is important that your prosthetic allows you to do the following things: ${q://QID101/ChoiceTextEntryValue}   How likely would you be to try this device if it could let you do these things?

- Very Unlikely
- Unlikely
- Maybe
- Likely
- Very Likely

Do you have any additional thoughts on this device? We are interested in understanding why you are interested or why you are not interested in trying this device.

Wireless Device Implanted in the Brain

Device Description: This device is an array of 100 tiny sensors in a package about the size of a baby aspirin. The sensors are placed 1.5 mm into the surface of the brain. The device senses brain cells firing and transmits this information to a prosthetic limb.

Medical Procedure: This device is put into the brain with a surgical procedure that requires opening the skull. Before surgery, hair around the incision area must be shaved. This procedure requires a hospital stay of about 3 days. The incision area will heal in about 2 weeks and the hair will regrow, making it unnoticeable.

Training Time: About 1 week.

Potential Risks: There is about a 3-5% risk of a minor problem (such as post-operative confusion) and a 1% risk of a major complication (such as a bleed or infection).

** This device is not currently available to the public.

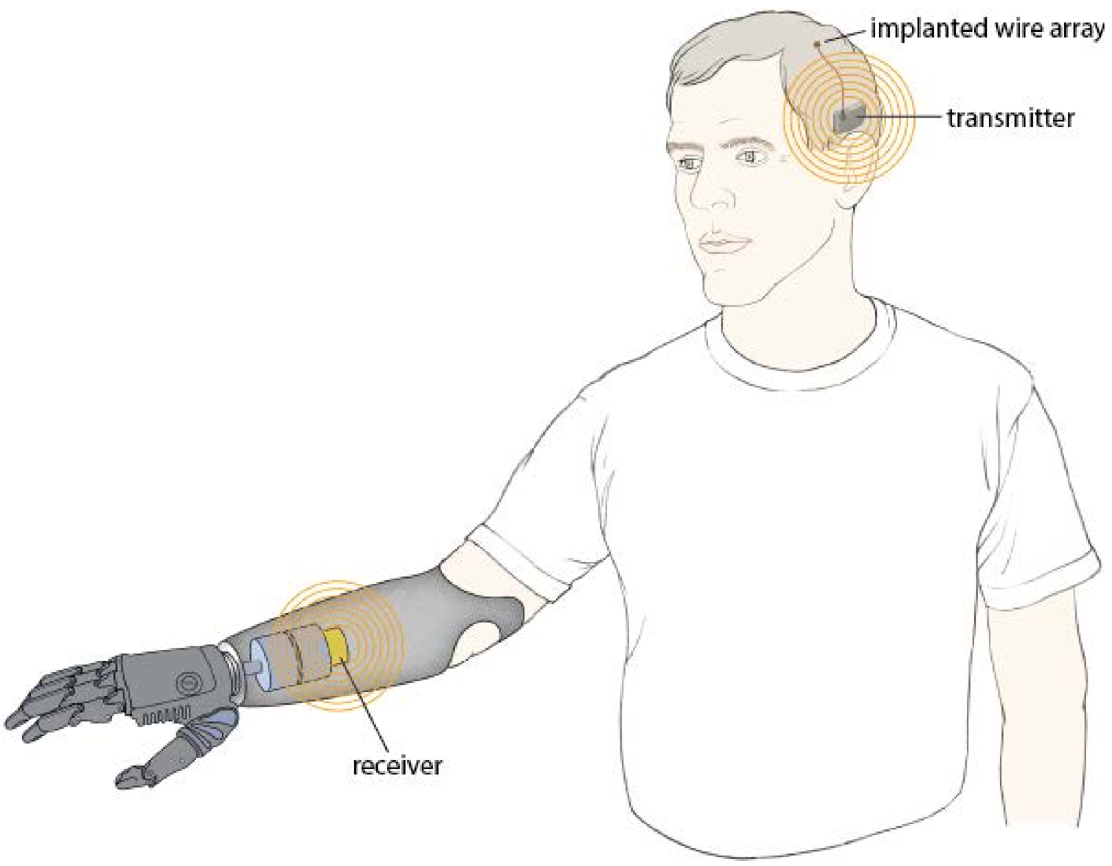

	With the procedure and risks in mind, how likely are you to have the device if it could let you:				
	Very Unlikely	Unlikely	Maybe	Likely	Very Likely
Open and close your hand slowly	❍	❍	❍	❍	❍
Do all the above AND rotate your wrist	❍	❍	❍	❍	❍
Do all the above AND move to any location in your workspace and perform a simple grasp	❍	❍	❍	❍	❍
Do all the above AND perform several types of grasps, in which you can control the amount of force used	❍	❍	❍	❍	❍
Do all the above AND perform tasks that require fine motor control (such as writing with a pen or typing)	❍	❍	❍	❍	❍
Do all the above AND have touch sensation in the missing limb	❍	❍	❍	❍	❍

You wrote that you think it is important that your prosthetic allows you to do the following things: ${q://QID101/ChoiceTextEntryValue} How likely would you be to try this device if it could let you do these things?

- ❍ Very Unlikely
- ❍ Unlikely
- ❍ Maybe
- ❍ Likely
- ❍ Very Likely

Do you have any additional thoughts on this device? We are interested in understanding why you are interested or why you are not interested in trying this device.

Thank you for your participation in this survey! We encourage you to include any additional comments in the space below.

In order to receive your $10 gift card, please email Susannah Engdahl at sengdahl@umich.edu with your name and mailing address. Your name and address will not be connected with your responses to this survey.

While providing your name and mailing address is optional, we will be unable to provide you with a gift card without this information.

**We are only able to send one gift card to each mailing address.
****If you do not live in the U.S., we will be unable to provide you with a gift card.**
