# Supplementary material for: Factors associated with interest in novel interfaces for upper limb prosthesis control
Source: PLoS One. 2017 Aug 2;12(8):e0182482. doi: 10.1371/journal.pone.0182482 (PMC5540477; doi:10.1371/journal.pone.0182482)

Contents

Factors 2
- Age 2
- Time Since Amputation 3
- Gender 4
- Level of Limb Loss 5
  - Combinations of limb loss levels for participants with bilateral limb loss 6
- Unilateral/Bilateral 7
- Cause of Limb Loss 8
- Side of Limb Loss 9
- Pain Frequency 10
- Prosthesis Necessity 11
- Prosthesis Use 12
- Prosthesis Type 13
- Prosthesis Satisfaction 14
- Myoelectric Use 15
- Functional Satisfaction 16
- Education 17
- Lower Limb Loss 18

Outcome measures 19
- MYO 19
- TMR 20
- PNI 21
- CI 22

Factors

Age

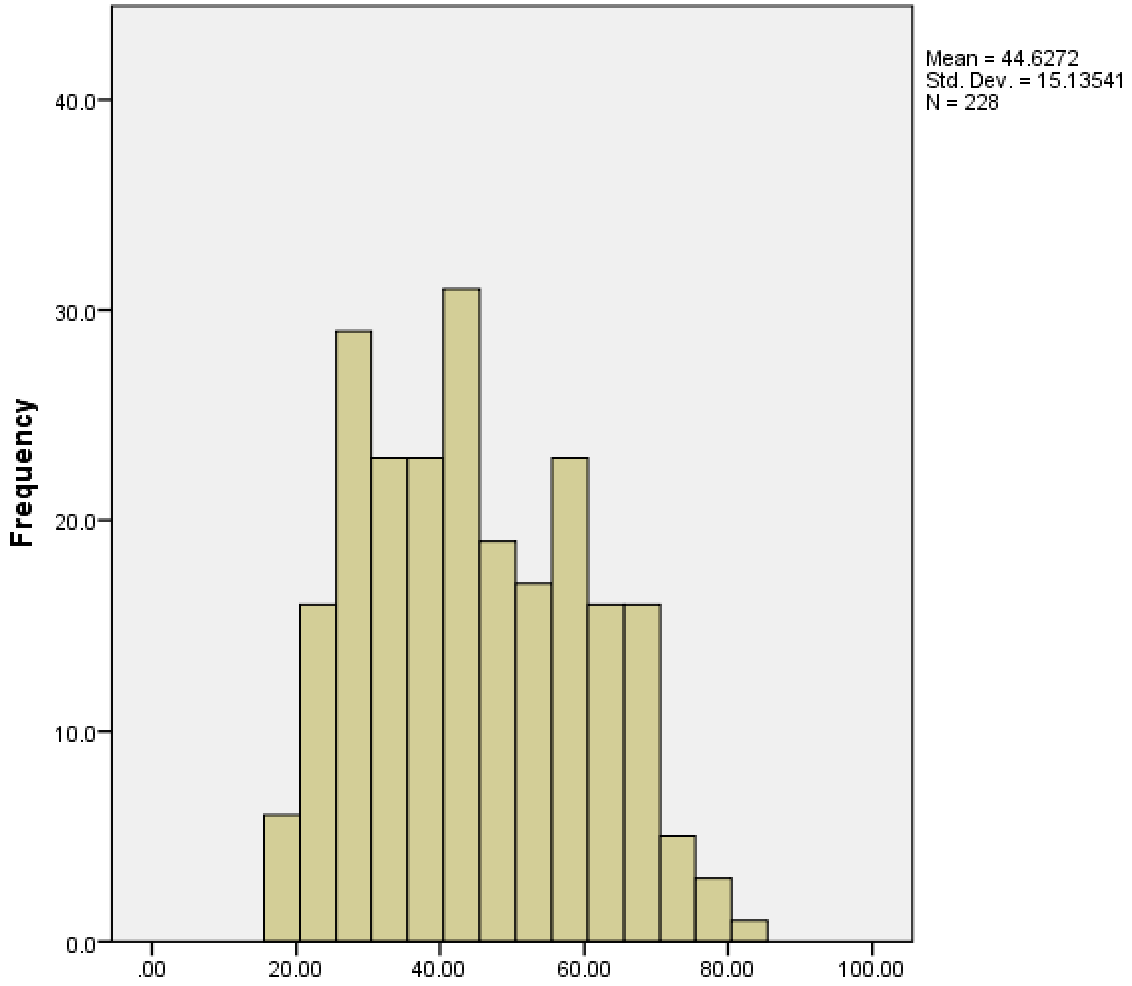

Mean = 44.6272
Std. Dev. = 15.13541
N = 228
Frequency
40.0
30.0
20.0
10.0
0.0
.00
20.00
40.00
60.00
80.00
100.00

Time Since Amputation

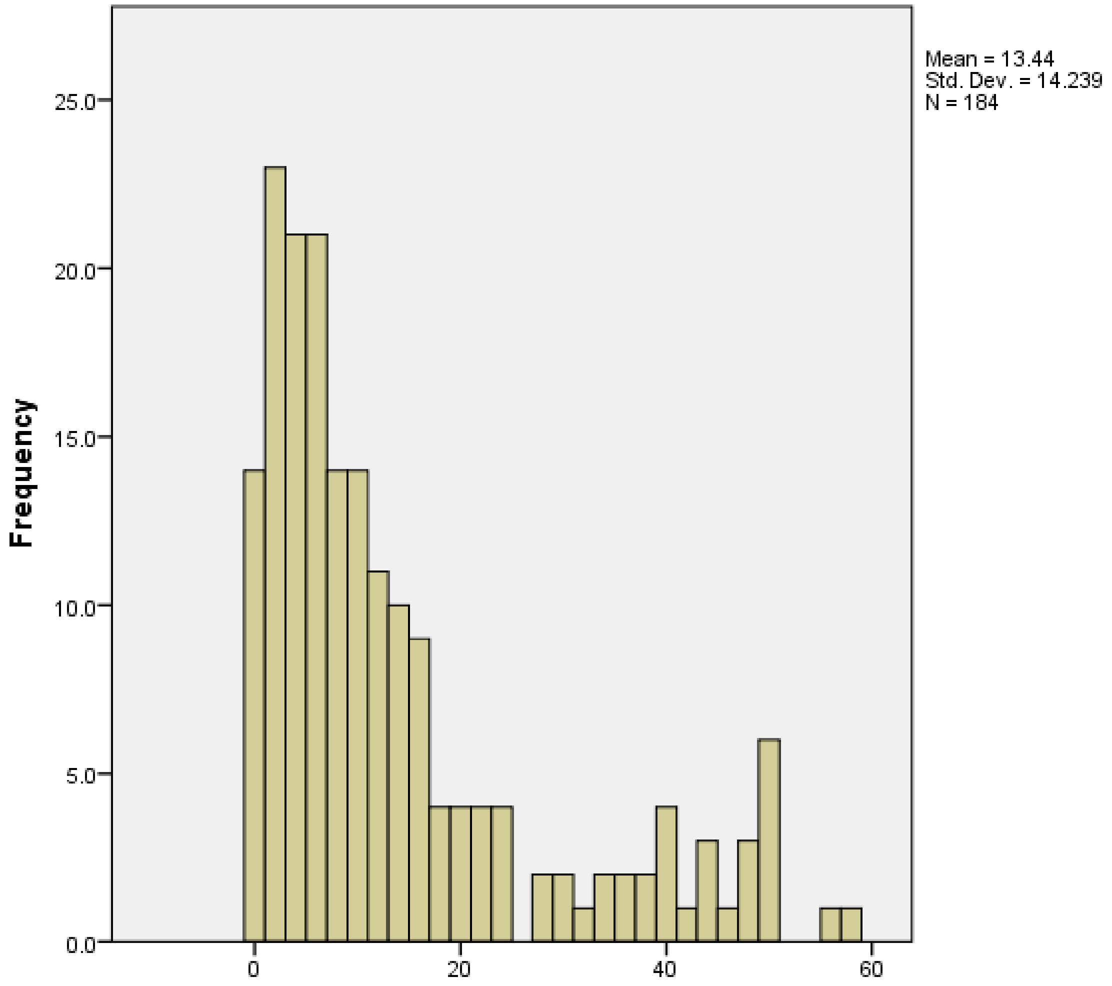

Gender

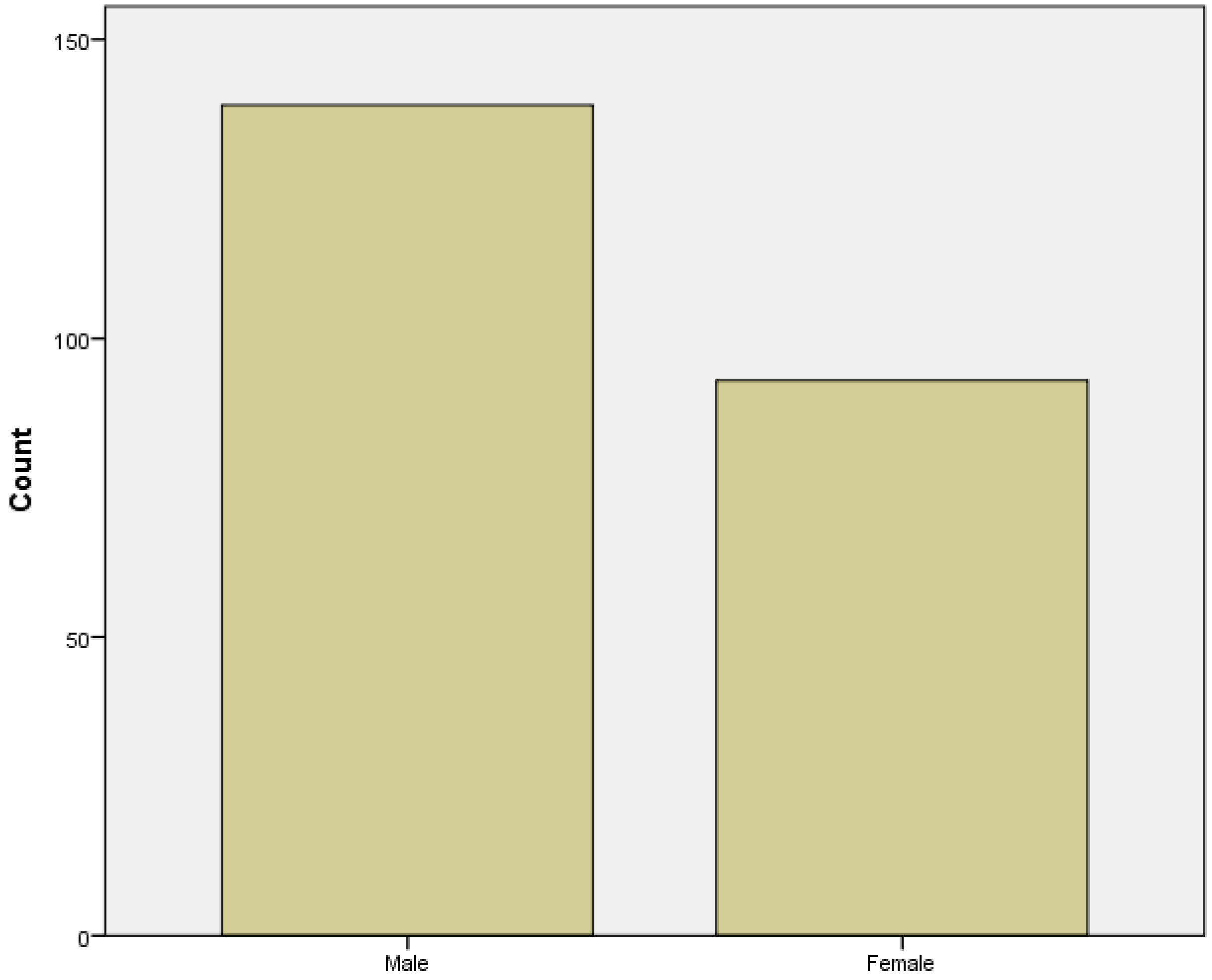

Level of Limb Loss

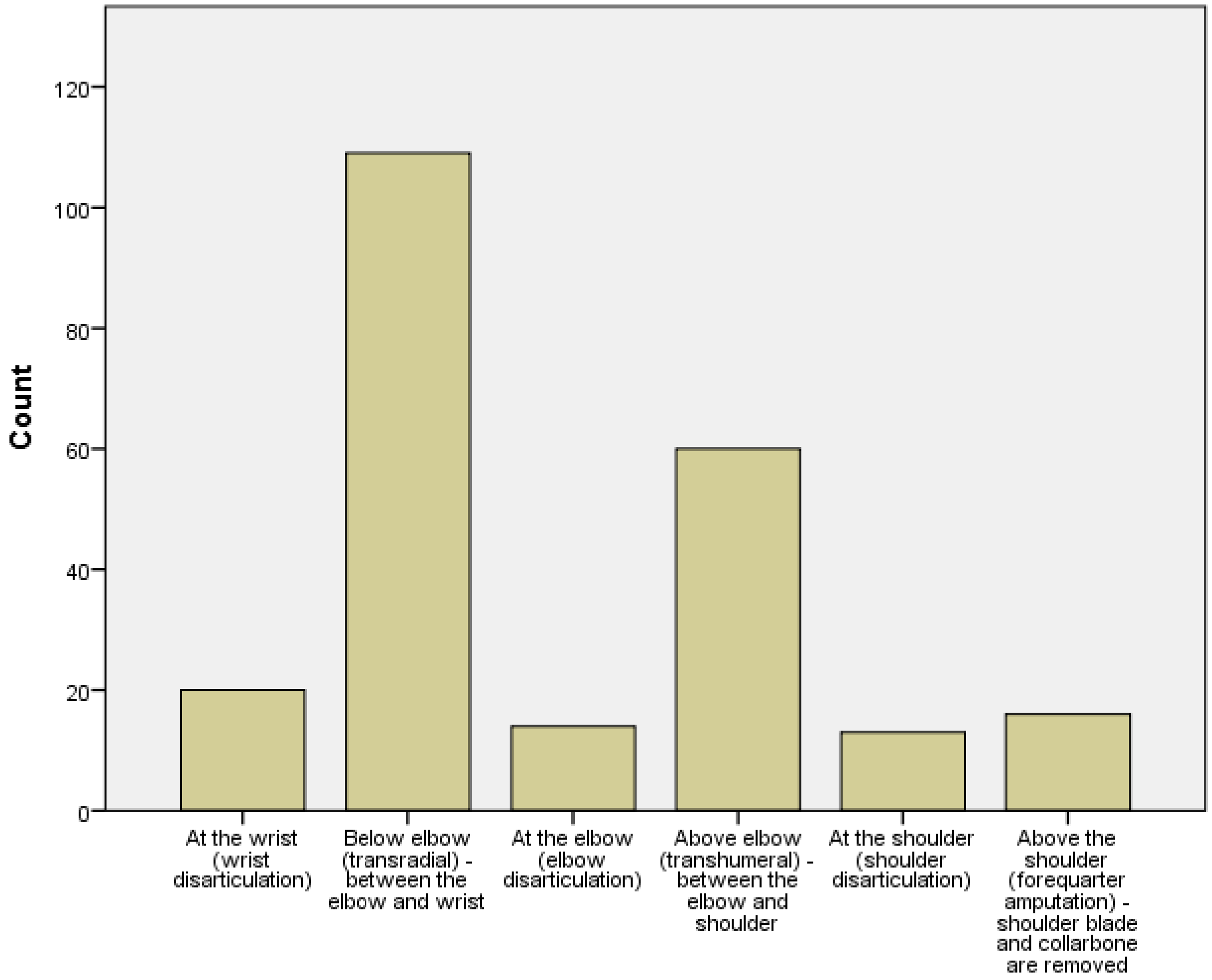

Combinations of limb loss levels for participants with bilateral limb loss

Right arm

Left arm

	**Partial hand**	**Wrist disarticulation**	**Transradial**	**Elbow disarticulation**	**Transhumeral**	**Shoulder disarticulation**	**Forequarter**
**Partial hand**					1		
**Wrist disarticulation**		2	4				
**Transradial**	3		11	1	1		1
**Elbow disarticulation**			3				
**Transhumeral**			1		3	1	1
**Shoulder disarticulation**						1	
**Forequarter**					1		

Unilateral/Bilateral

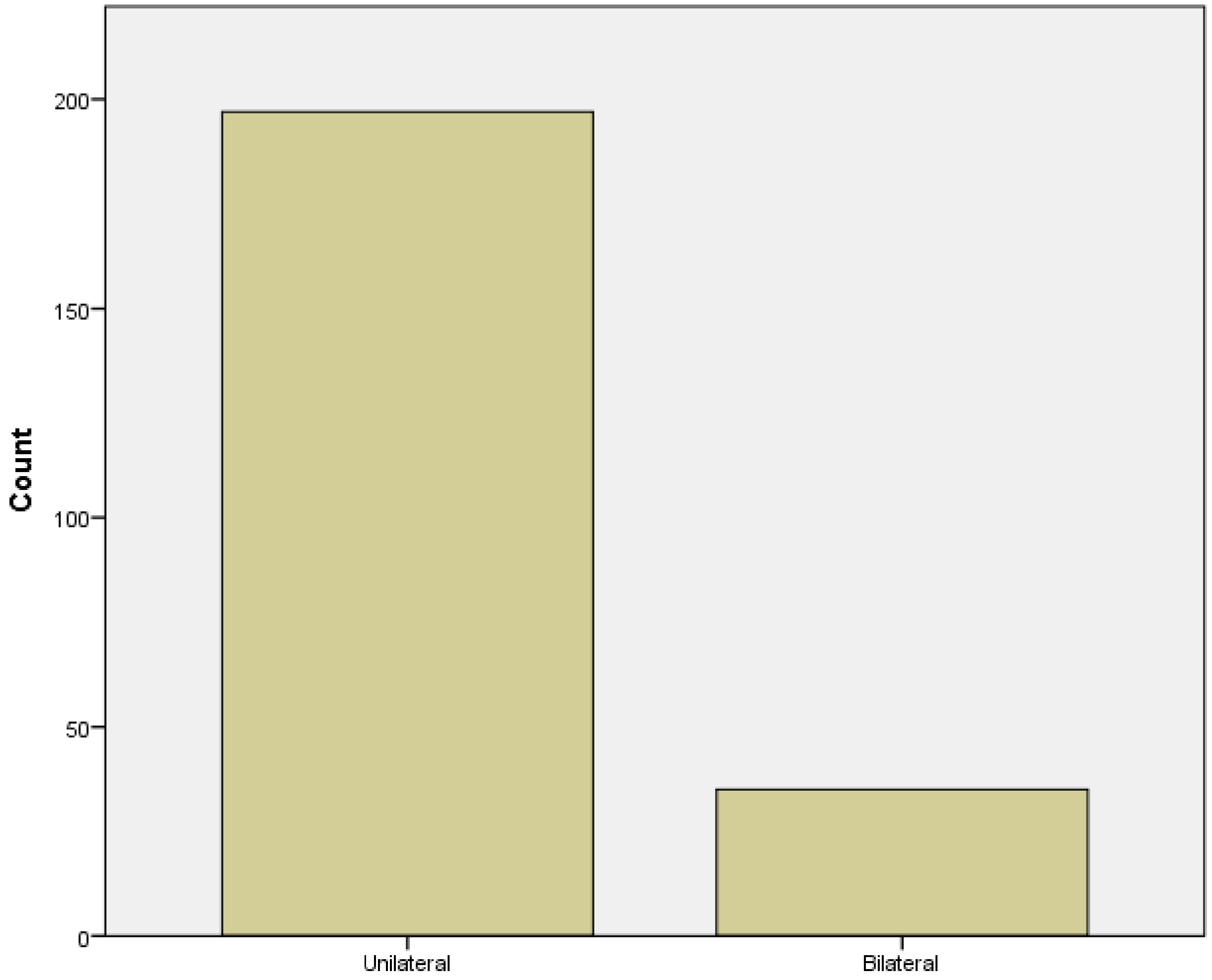

Cause of Limb Loss

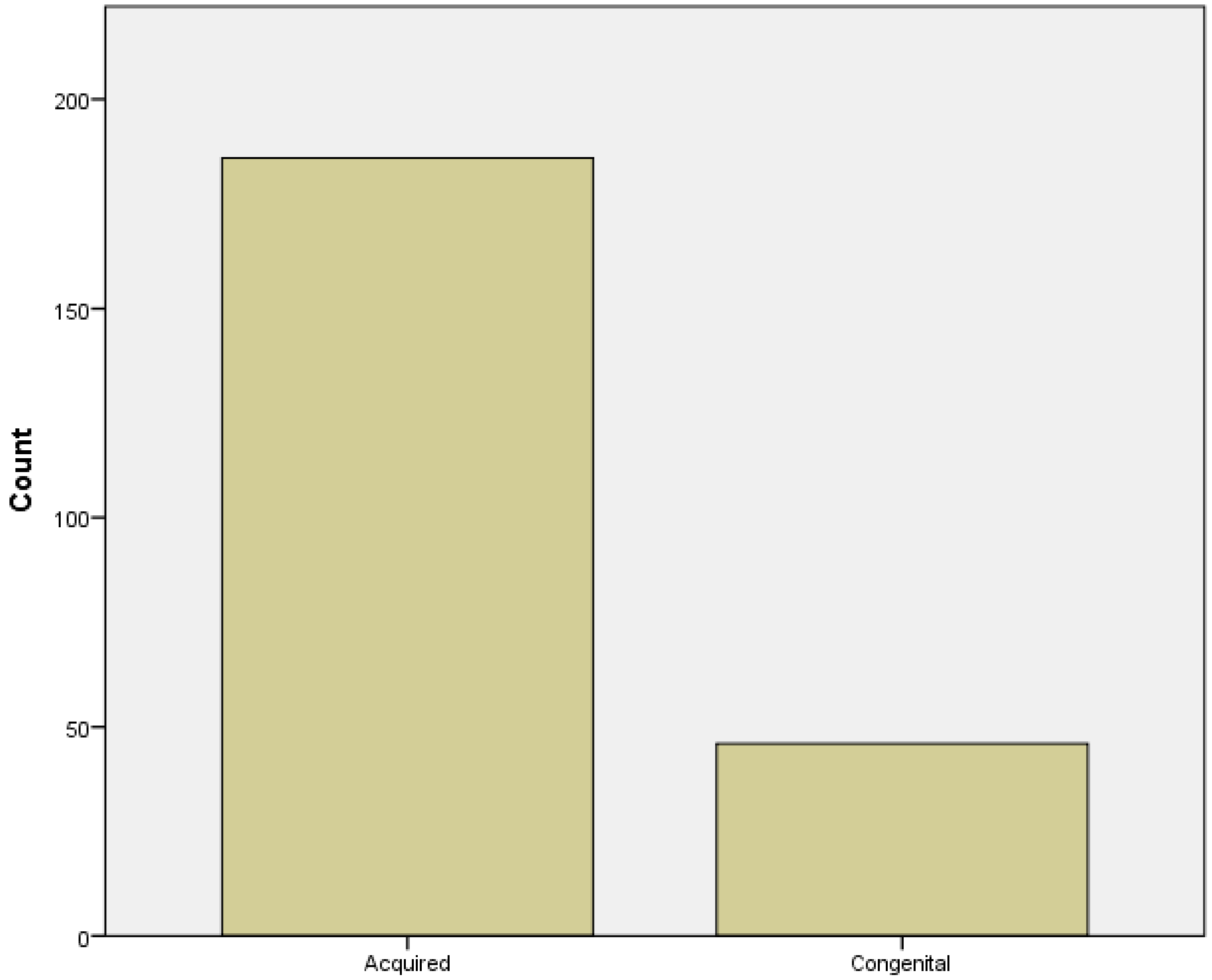

Side of Limb Loss

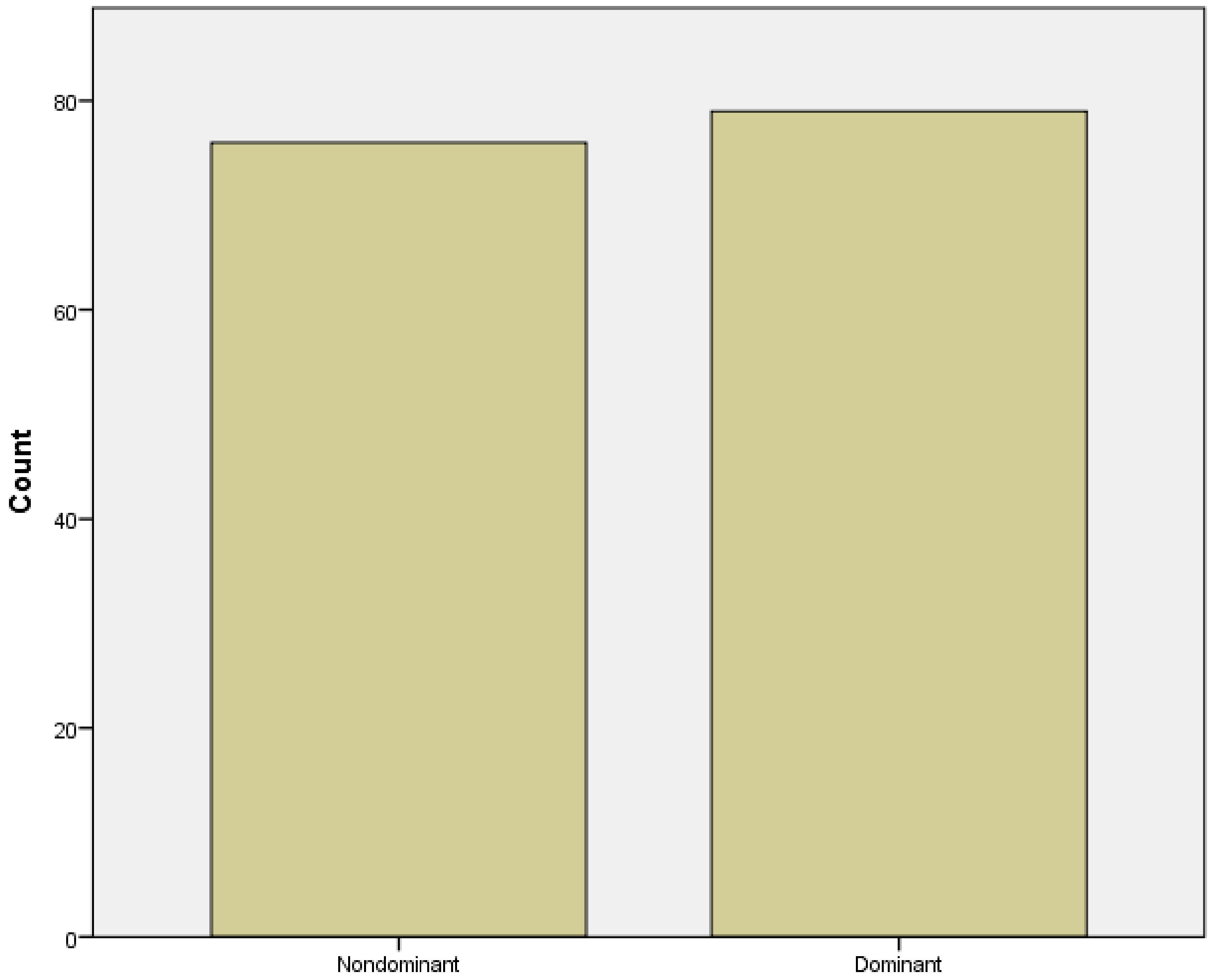

Pain Frequency

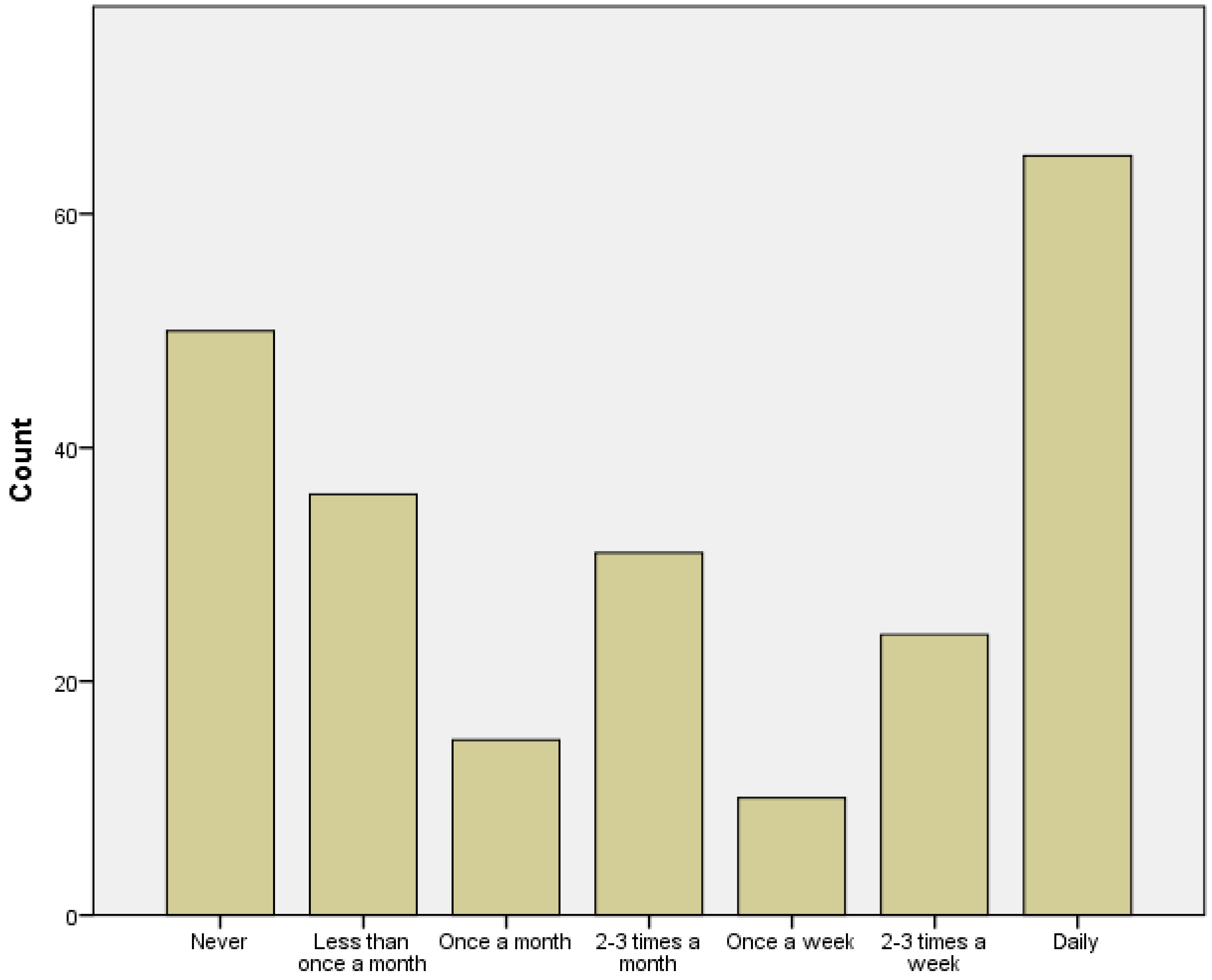

Prosthesis Necessity

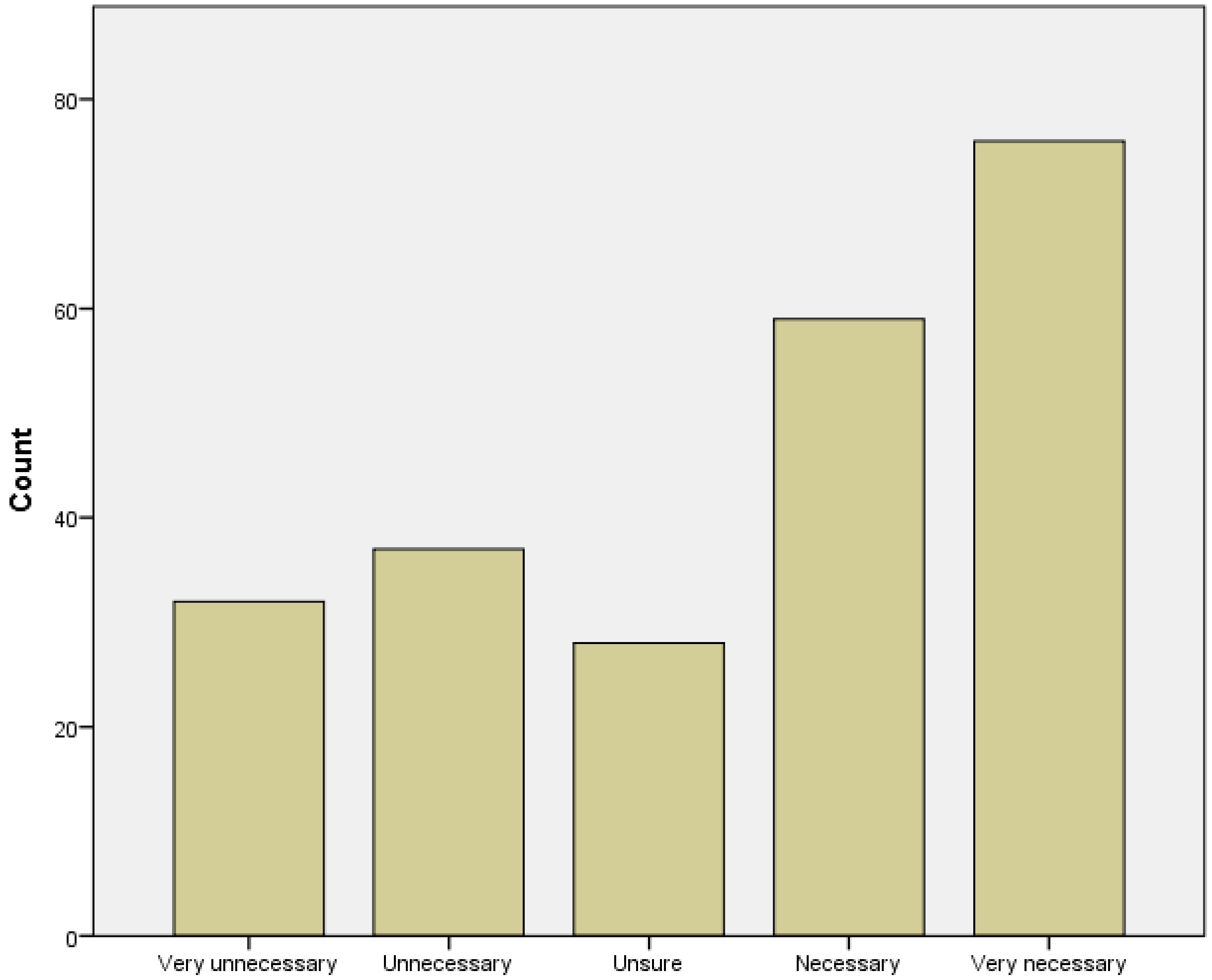

Prosthesis Use

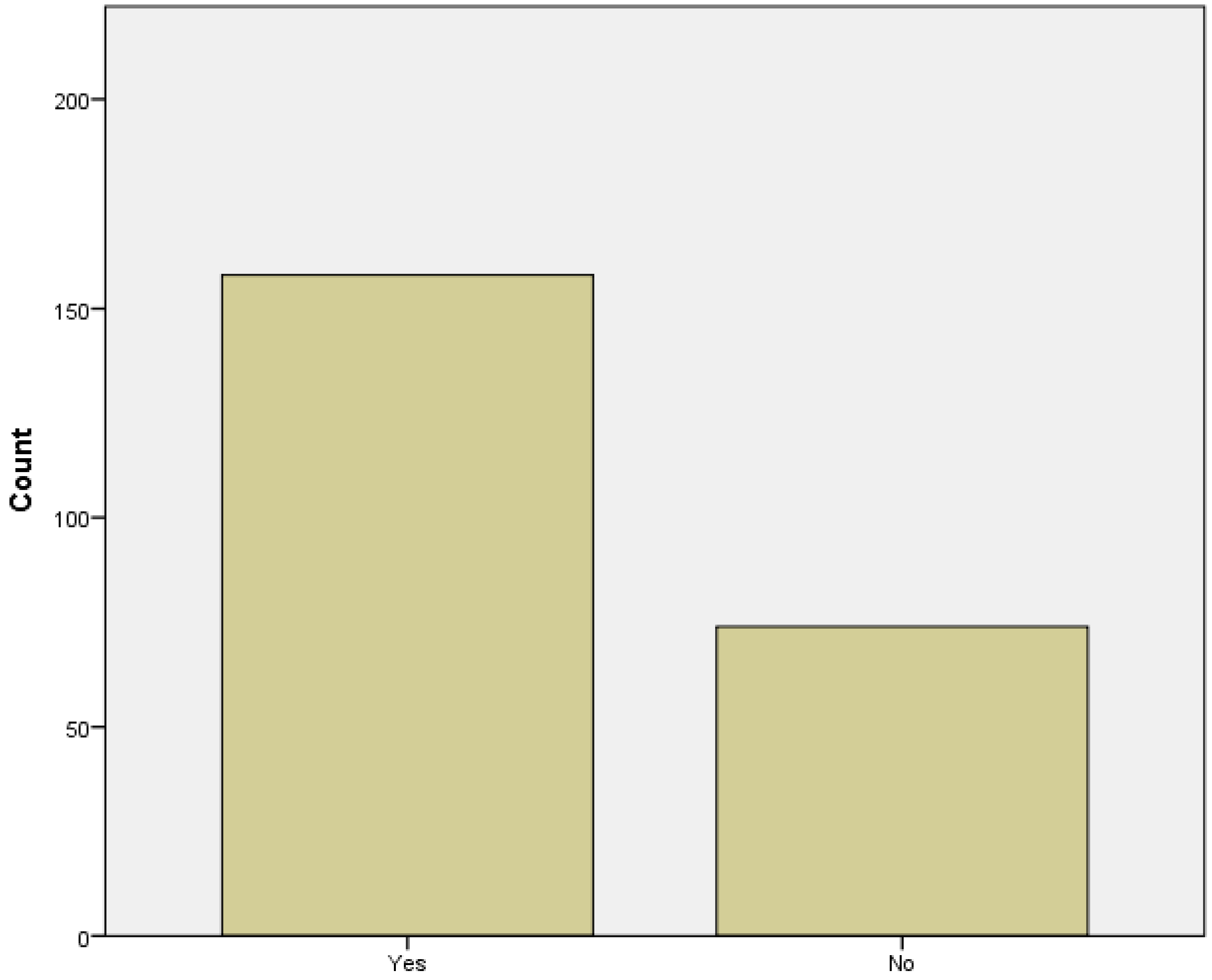

Prosthesis Type

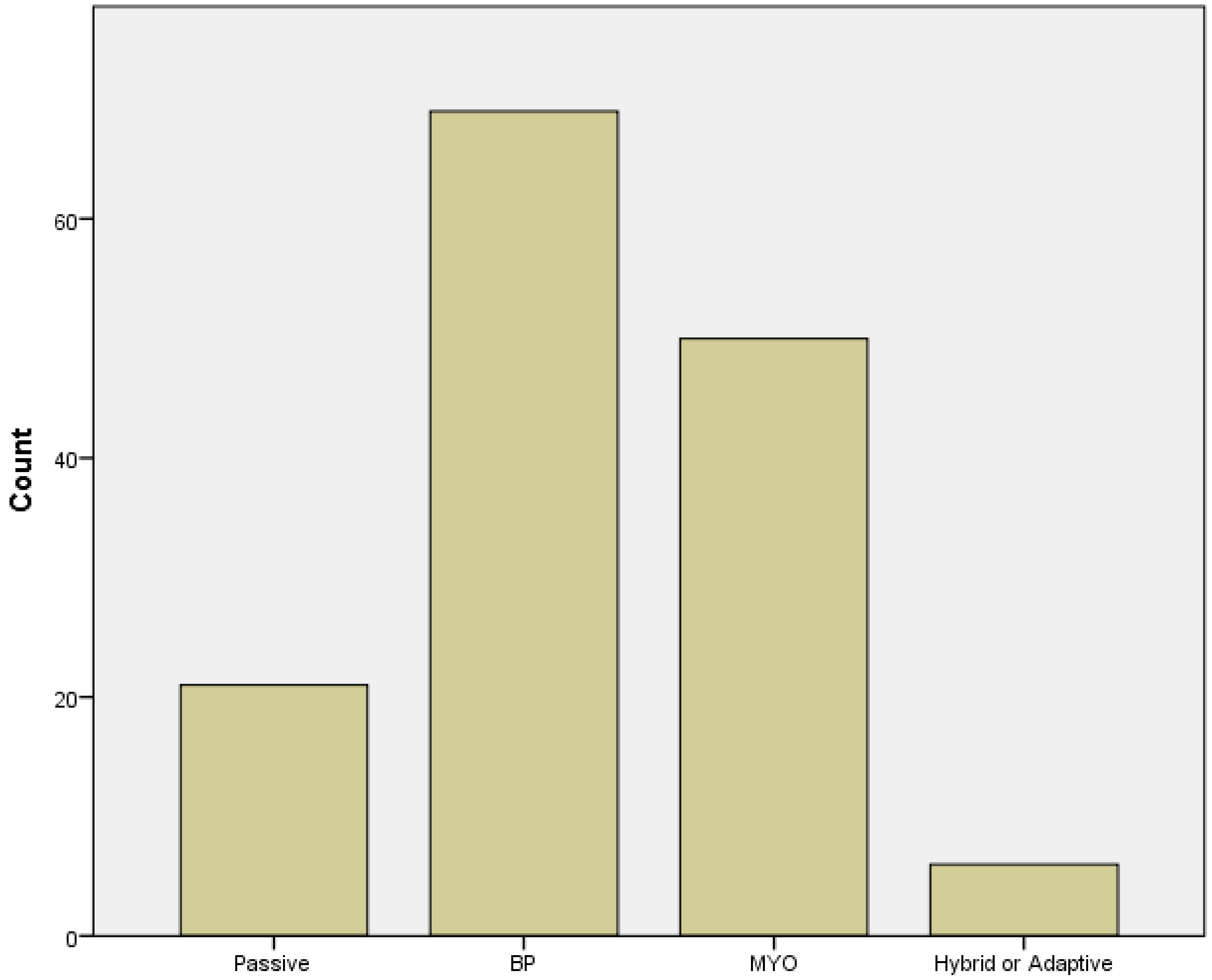

Prosthesis Satisfaction

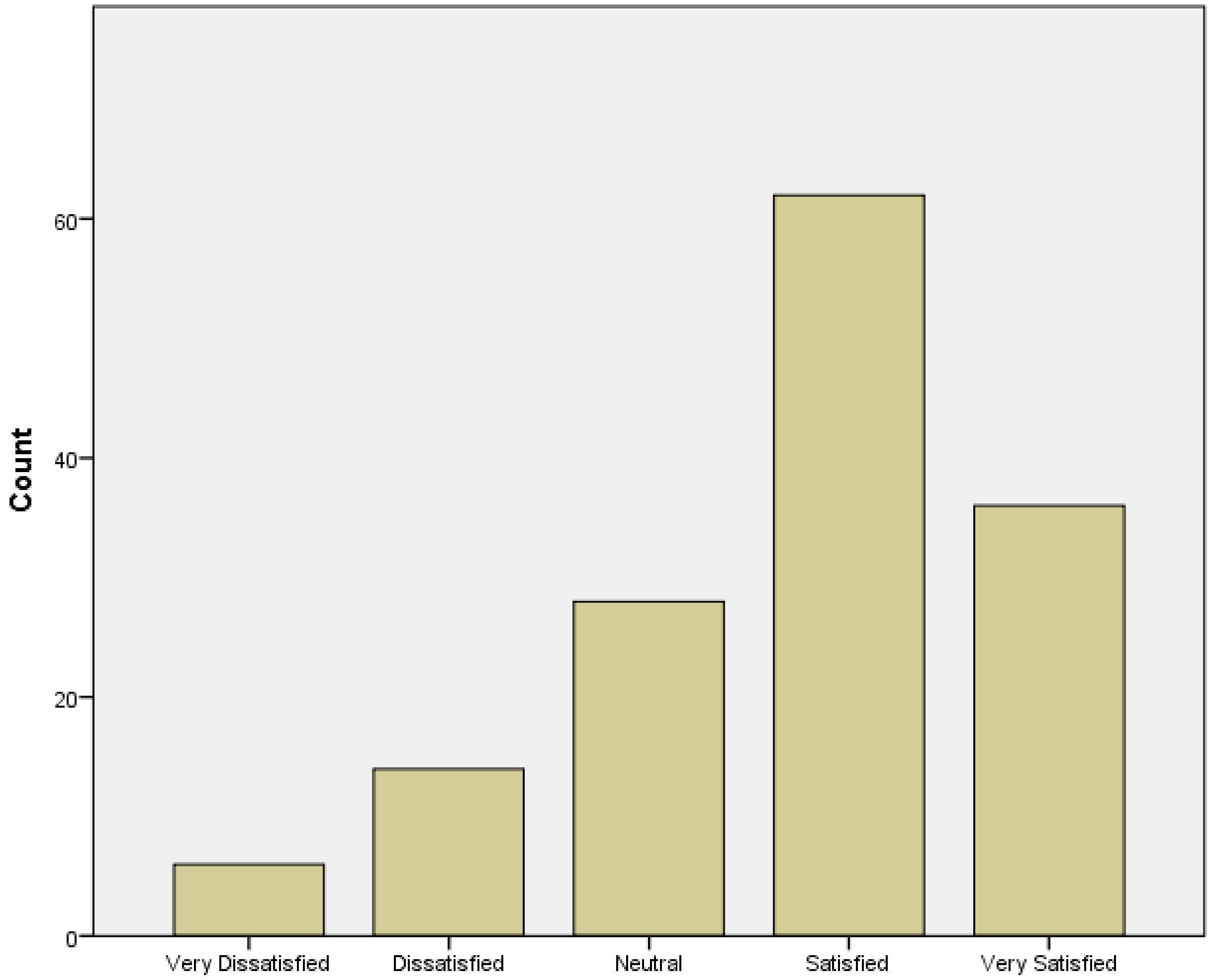

Myoelectric Use

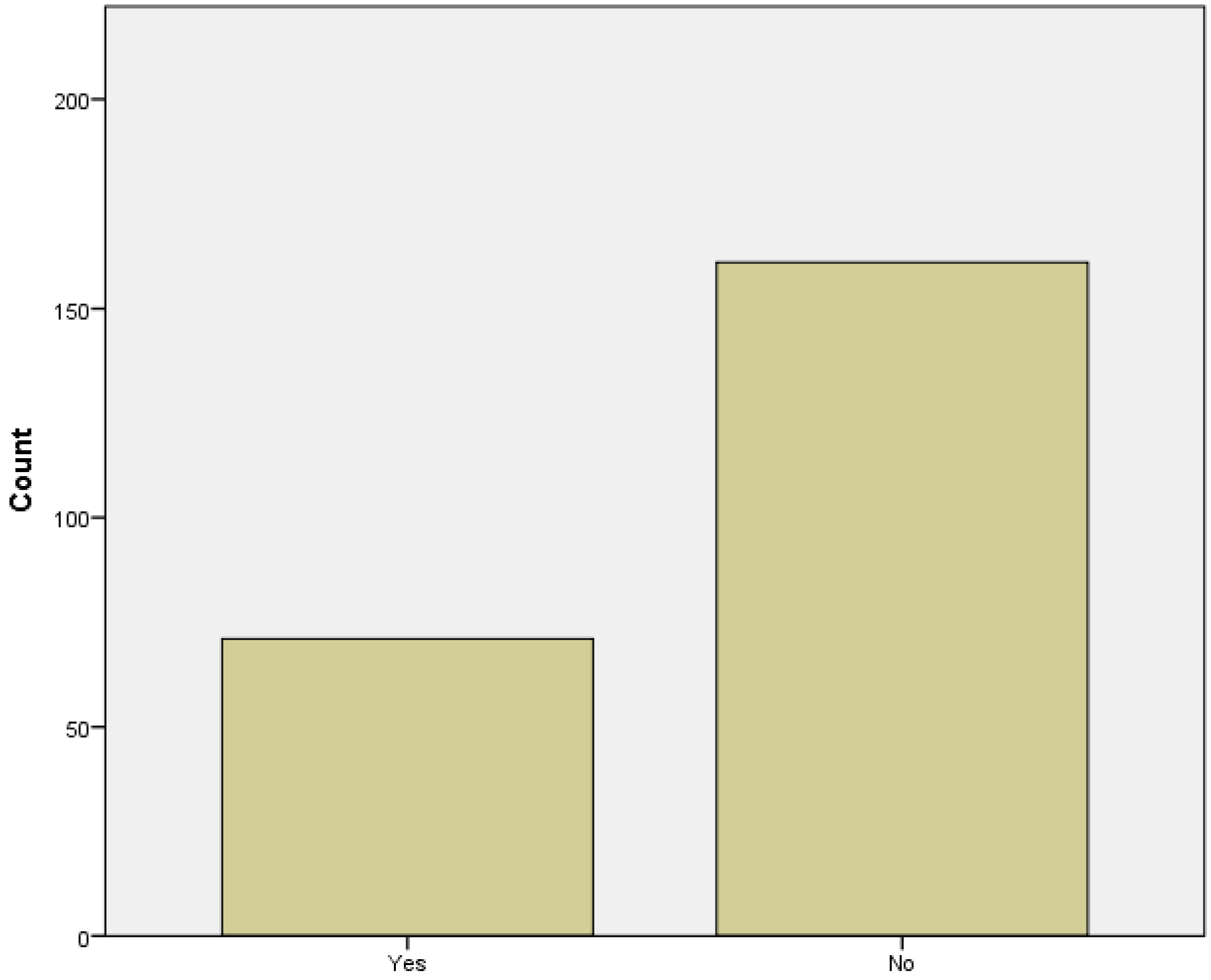

Functional Satisfaction

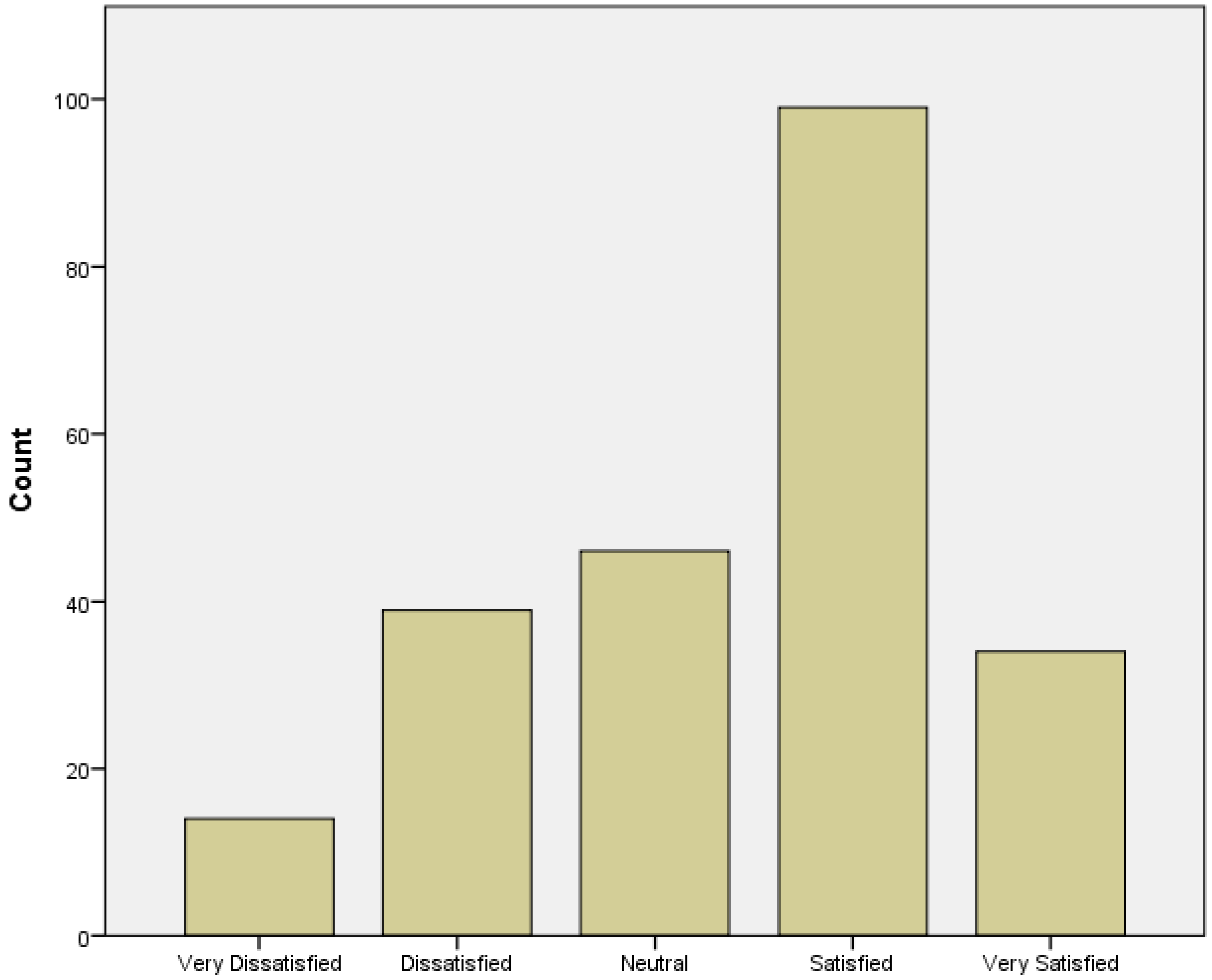

Education

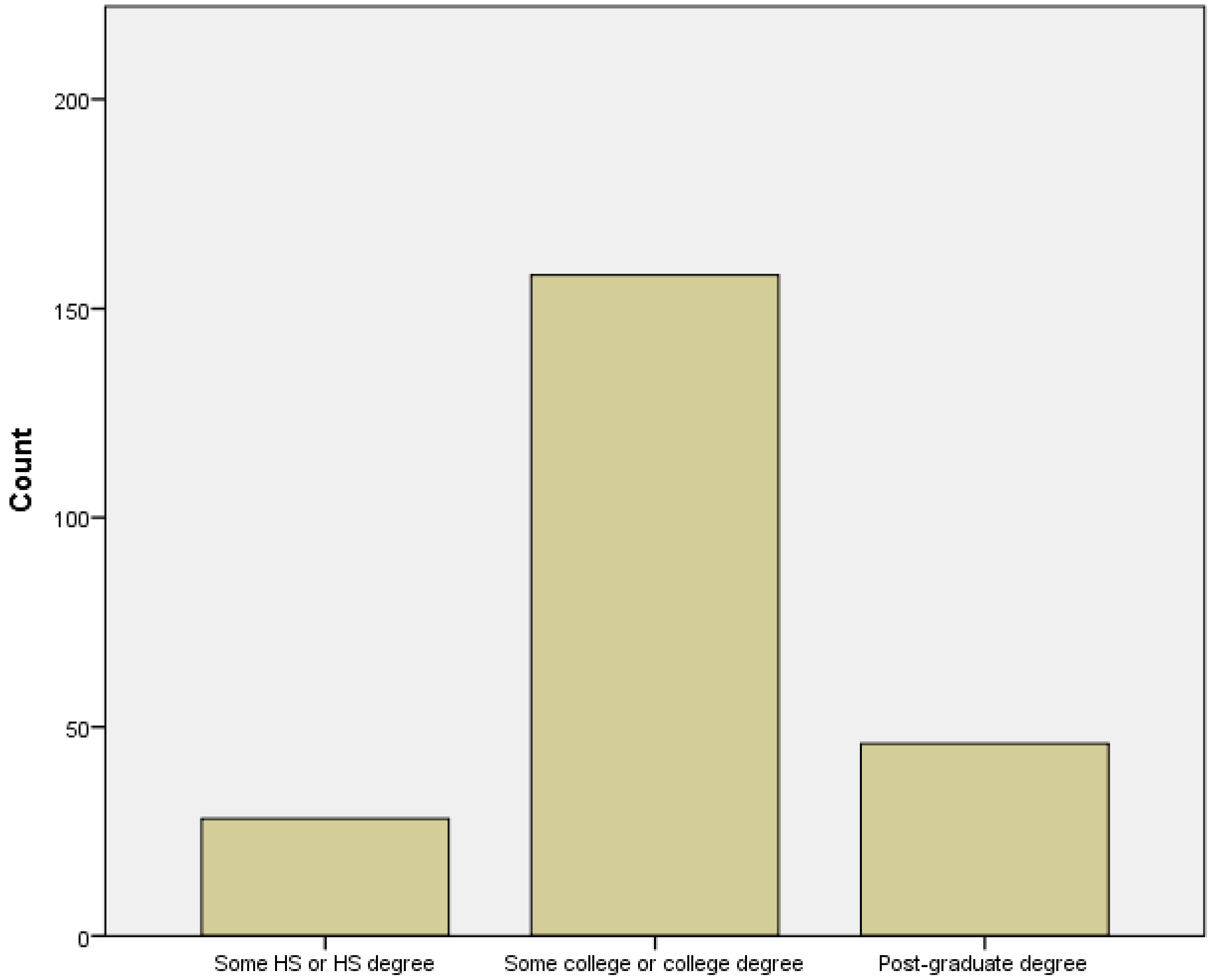
Count
200
150
100
50
0
Some HS or HS degree
Some college or college degree
Post-graduate degree

Lower Limb Loss

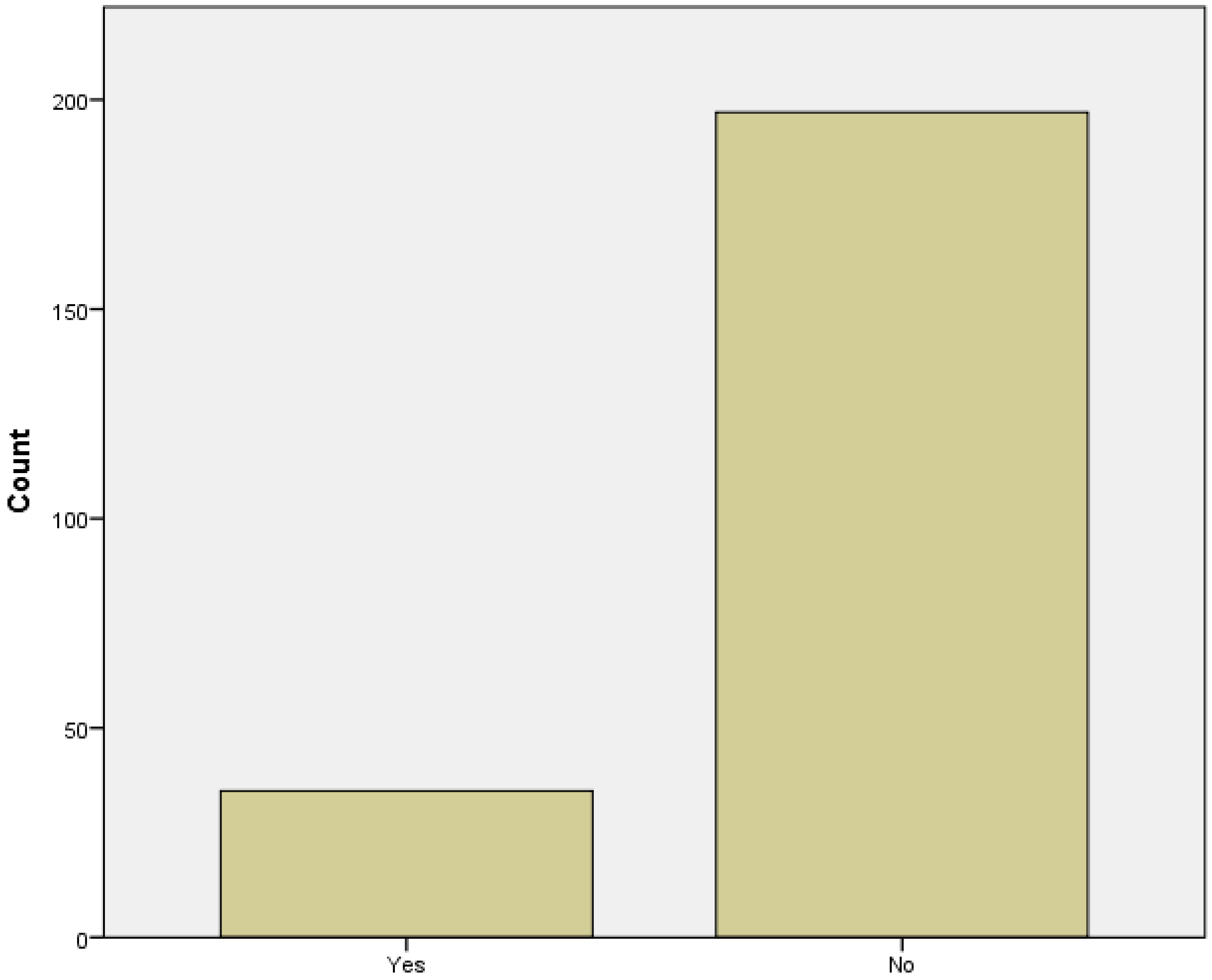

Outcome measures

MYO

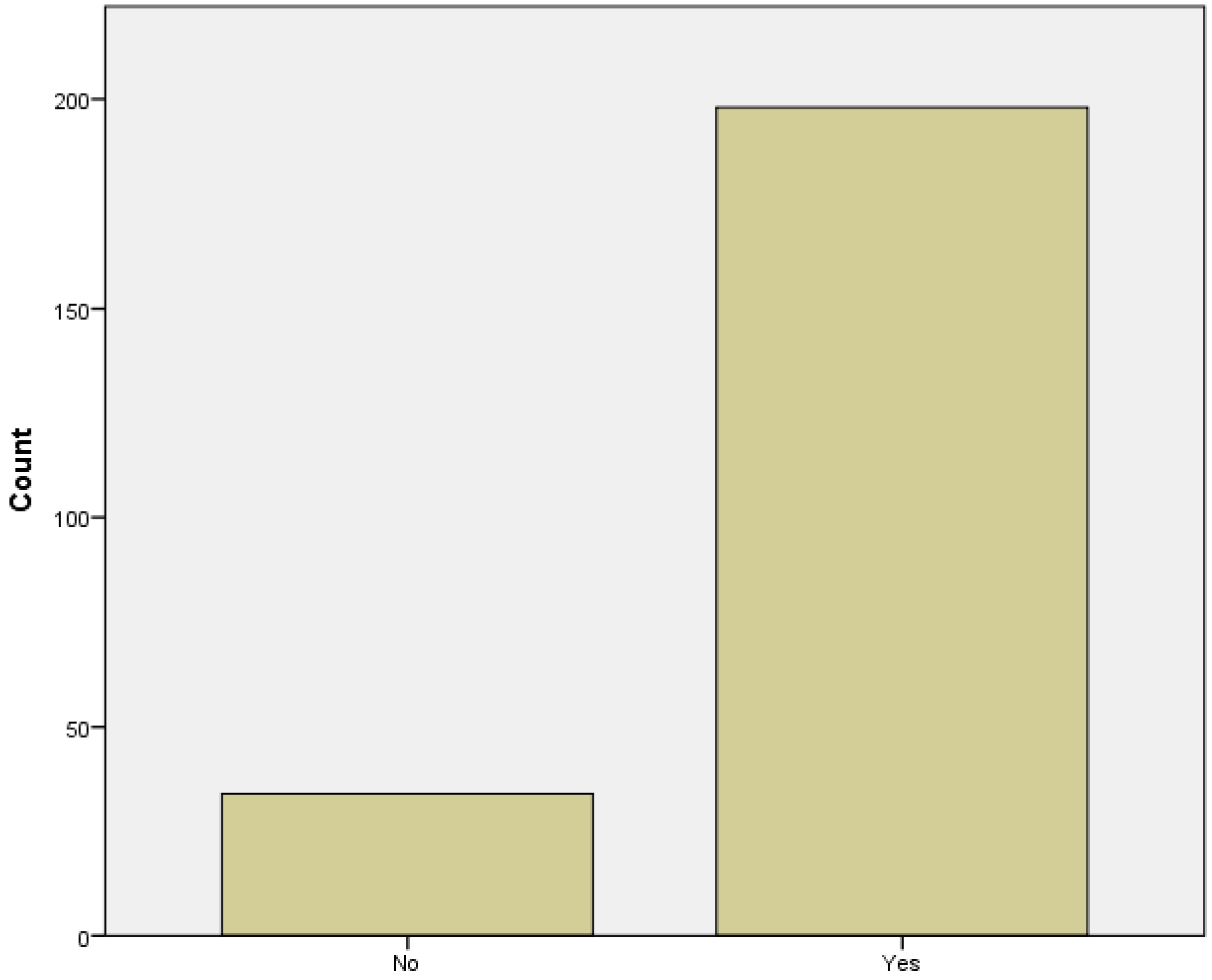

TMR

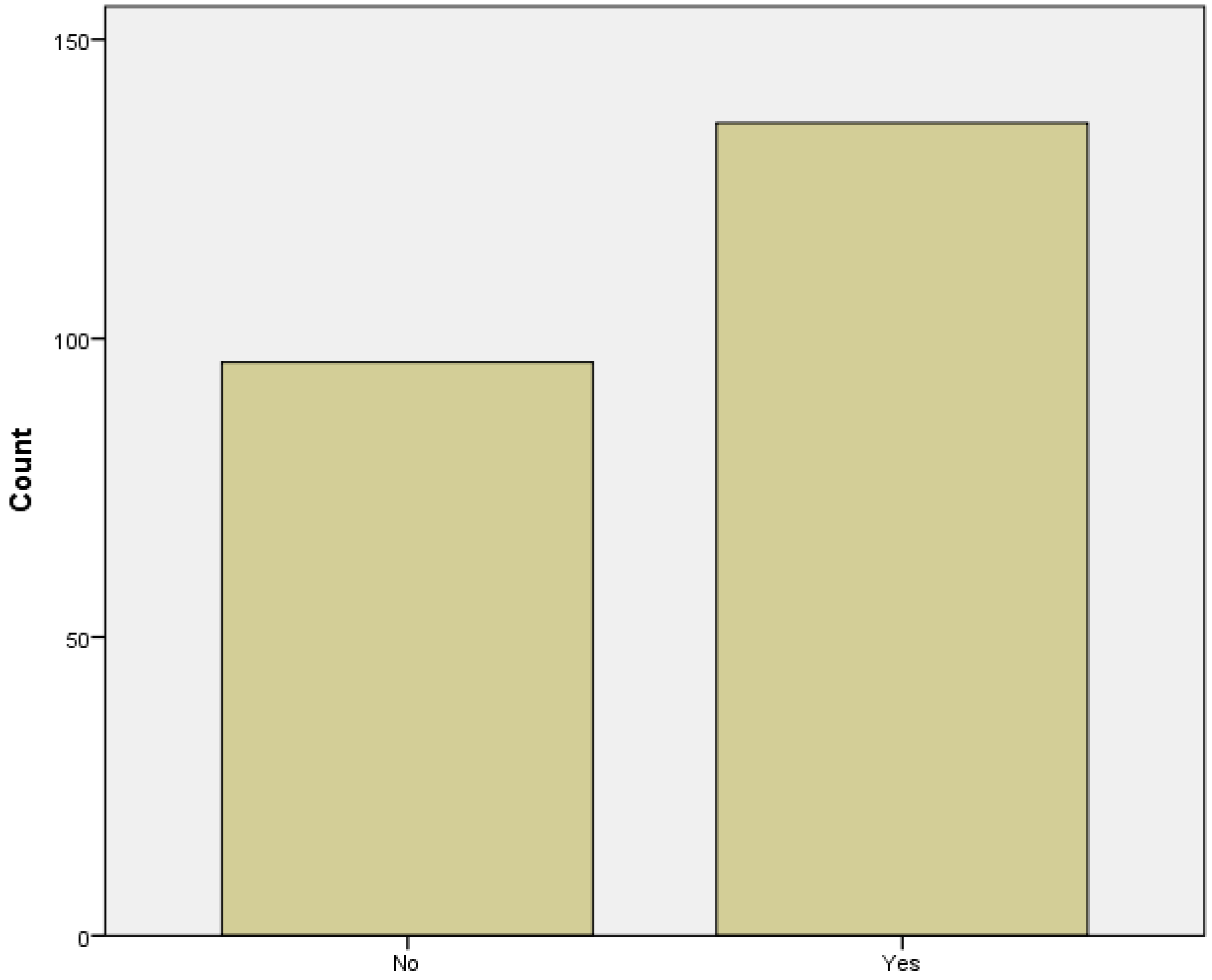

PNI

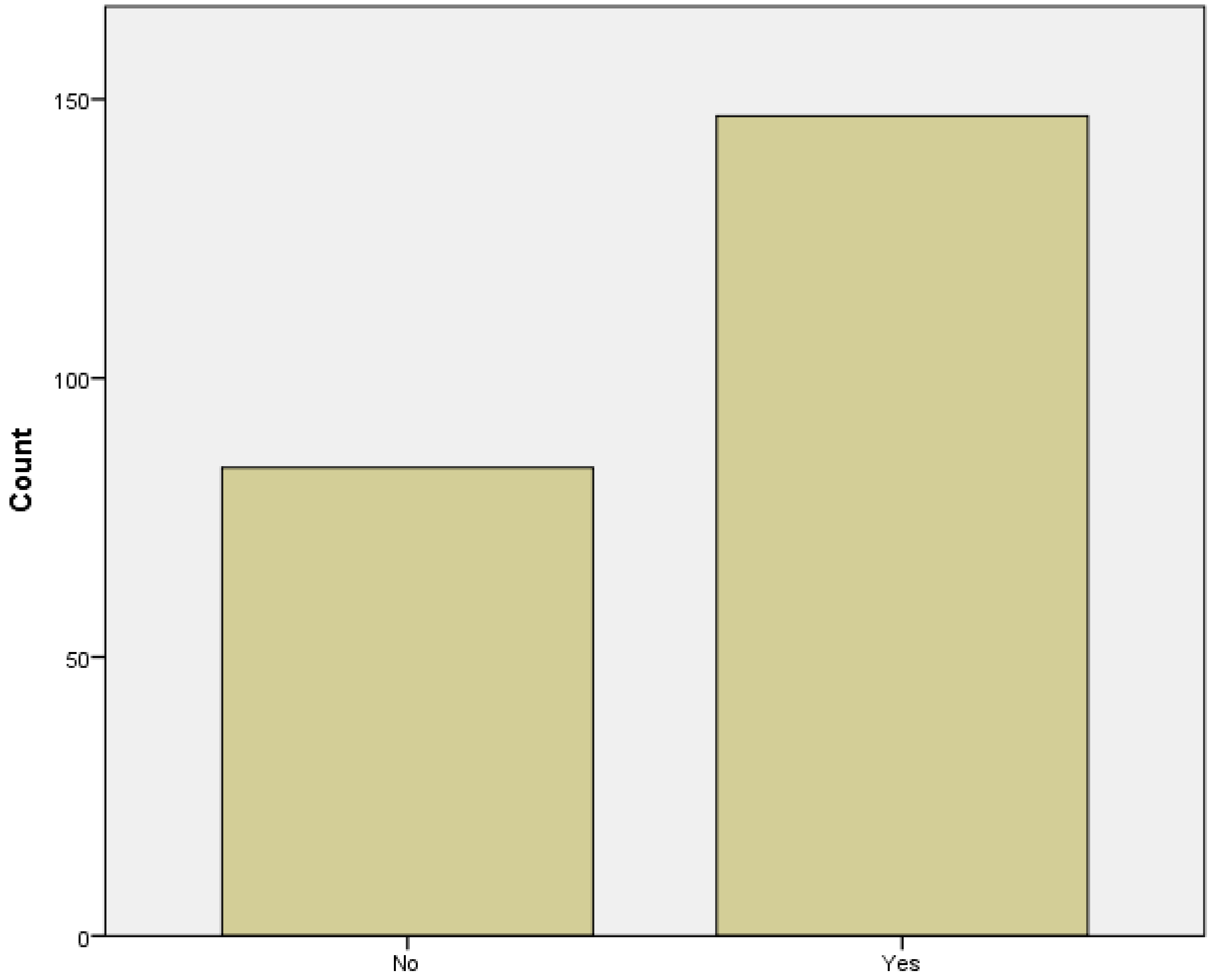

CI

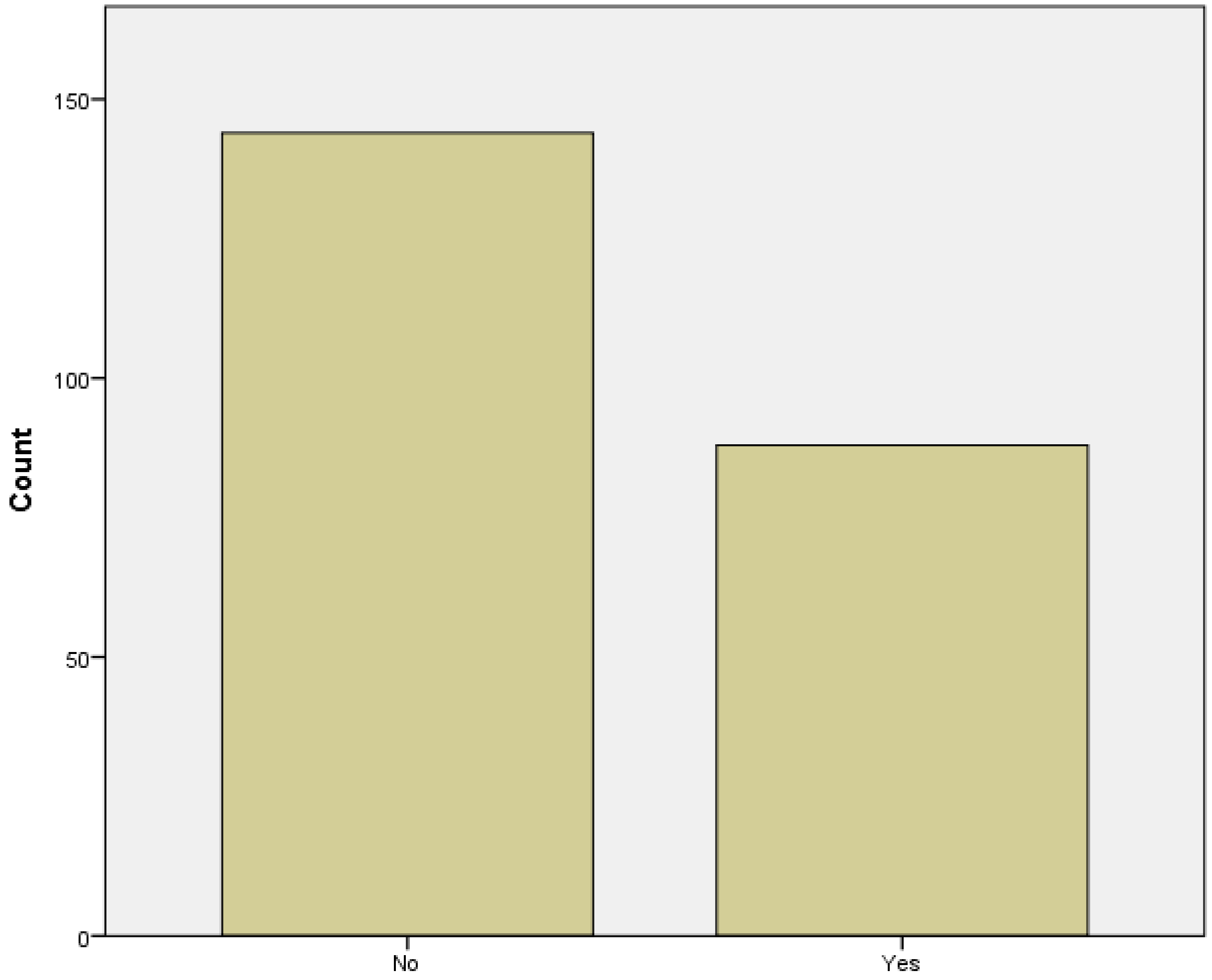

Supplement: S2 Appendix — (PDF) [file pone.0182482.s002.pdf]
