# Supplementary material for: Factors associated with interest in novel interfaces for upper limb prosthesis control
Source: PLoS One. 2017 Aug 2;12(8):e0182482. doi: 10.1371/journal.pone.0182482 (PMC5540477; doi:10.1371/journal.pone.0182482)
Supplement: S3 Appendix — (PDF) [file pone.0182482.s003.pdf]

Contents

Models for participants with acquired limb loss only (n=186) 2

Logistic regression model for MYO. 2

Logistic regression model for TMR. 2

Logistic regression model for PNI. 3

Logistic regression model for CI. 3

Area under ROC curves. 3

Models for participants who use a prosthesis only (n=158) 4

Logistic regression model for MYO. 4

Logistic regression model for TMR. 4

Logistic regression model for PNI. 5

Logistic regression model for CI. 5

Area under ROC curves. 6

<u>Models for participants with acquired limb loss only (n=186)</u>

Logistic regression model for MYO.

	**B [a]**	**S.E.**	**p**	**Odds Ratio**	**95% CI for Odds Ratio**	**Reference Category**
Time Since Amputation	-0.041	0.019	**0.032**	0.960	[0.92, 1.00]	n/a
Unilateral/Bilateral	2.012	0.623	**0.001**	7.476	[2.20, 25.35]	Bilateral
Pain Frequency	0.036	0.133	0.788	1.036	[0.80, 1.34]	n/a
Prosthesis Necessity	0.193	0.199	0.333	1.212	[0.82, 1.79]	n/a
Prosthesis Use	0.652	0.639	0.308	1.920	[0.55, 6.72]	No Use
Myoelectric Use	2.341	1.107	**0.034**	10.394	[1.19, 90.93]	No Use
Functional Satisfaction	-0.140	0.265	0.599	0.870	[0.52, 1.46]	n/a
(Constant)	0.085	1.496	0.955	-	-	-

Model χ^2 (7) = 30.0, $p < 0.001$.
[a] unstandardized regression coefficient

Logistic regression model for TMR.

	**B [a]**	**S.E.**	**p**	**Odds Ratio**	**95% CI for Odds Ratio**	**Reference Category**
Age	-0.034	0.014	**0.012**	0.966	[0.94, 0.99]	n/a
Time Since Amputation	-0.037	0.016	**0.020**	0.964	[0.93, 0.99]	n/a
Gender	0.832	0.406	**0.040**	2.298	[1.04, 5.09]	Female
Unilateral/Bilateral	1.389	0.467	**0.003**	4.012	[1.61, 10.01]	Bilateral
Pain Frequency	-0.064	0.091	0.482	0.938	[0.78, 1.12]	n/a
Functional Satisfaction	-0.047	0.169	0.784	0.955	[0.68, 1.33]	n/a
Education	-0.902	0.337	**0.007**	0.406	[0.21, 0.78]	n/a
(Constant)	3.349	1.291	0.009	-	-	-

Model χ^2 (7) = 45.9, $p < 0.001$.
[a] unstandardized regression coefficient

Logistic regression model for PNI.

	B [a]	S.E.	p	Odds Ratio	95% CI for Odds Ratio	Reference Category
Age	-0.039	0.015	**0.008**	0.962	[0.93, 0.99]	n/a
Time Since Amputation	-0.036	0.016	**0.021**	0.964	[0.93, 0.99]	n/a
Gender	1.478	0.440	**0.001**	4.384	[1.85, 10.38]	Female
Unilateral/Bilateral	1.743	0.484	**0.000**	5.713	[2.21, 14.74]	Bilateral
Pain Frequency	-0.022	0.096	0.815	0.978	[0.81, 1.18]	n/a
Functional Satisfaction	-0.318	0.185	0.086	0.728	[0.51, 1.05]	n/a
(Constant)	2.054	1.165	0.078	-	-	-

Model χ^2 (6) = 52.4, p < 0.001.
[a] unstandardized regression coefficient

Logistic regression model for CI.

	B [a]	S.E.	p	Odds Ratio	95% CI for Odds Ratio	Reference Category
Age	-0.052	0.013	**0.000**	0.950	[0.92, 0.98]	n/a
Time Since Amputation	-0.018	0.016	0.259	0.982	[0.95, 1.01]	n/a
Gender	0.786	0.387	**0.042**	2.194	[1.03, 4.68]	Female
Unilateral/Bilateral	1.156	0.514	**0.024**	3.178	[1.16, 8.70]	Bilateral
Pain Frequency	-0.048	0.087	0.578	0.953	[0.80, 1.13]	n/a
Prosthesis Necessity	0.176	0.127	0.167	1.193	[0.93, 1.53]	n/a
Education	-0.638	0.314	**0.042**	0.528	[0.29, 0.98]	n/a
(Constant)	1.699	1.239	0.170	-	-	-

Model χ^2 (7) = 45.0, p < 0.001.
[a] unstandardized regression coefficient

Area under ROC curves.

	AUC	p	95% CI
MYO	0.821	< 0.001	[0.722, 0.920]
TMR	0.770	< 0.001	[0.698, 0.842]
PNI	0.810	< 0.001	[0.740, 0.880]
CI	0.773	< 0.001	[0.706, 0.839]

Models for participants who use a prosthesis only (n=158)

Logistic regression model for MYO.

	**B [a]**	**S.E.**	**p**	**Odds Ratio**	**95% CI for Odds Ratio**	**Reference Category**
Unilateral/Bilateral	1.533	0.766	**0.045**	4.634	[1.03, 20.78]	Bilateral
Cause of Limb Loss	0.669	0.891	0.453	1.952	[0.34, 11.18]	Congenital
Pain Frequency	0.314	0.177	0.077	1.369	[0.97, 1.94]	n/a
Prosthesis Necessity	0.278	0.271	0.305	1.321	[0.78, 2.25]	n/a
Prosthesis Satisfaction	-1.168	0.564	**0.038**	0.311	[0.10, 0.94]	n/a
Myoelectric Use	2.661	1.118	**0.017**	14.316	[1.60, 127.98]	No Use
Functional Satisfaction	0.341	0.399	0.394	1.406	[0.64, 3.08]	n/a
(Constant)	1.234	2.503	0.622	-	-	-

Model χ^2 (7) = 33.3, p > 0.001.
[a] unstandardized regression coefficient

Logistic regression model for TMR.

	**B [a]**	**S.E.**	**p**	**Odds Ratio**	**95% CI for Odds Ratio**	**Reference Category**
Age	-0.039	0.015	**0.008**	0.962	[0.93, 0.99]	
Gender	0.546	0.496	0.272	1.726	[0.65, 4.57]	Female
Unilateral/Bilateral	1.343	0.546	**0.014**	3.829	[1.31, 11.16]	Bilateral
Cause of Limb Loss	0.989	0.625	0.114	2.689	[0.79, 9.15]	Congenital
Pain Frequency	0.086	0.098	0.377	1.090	[0.90, 1.32]	n/a
Prosthesis Satisfaction	-0.153	0.243	0.527	0.858	[0.53, 1.38]	n/a
Functional Satisfaction	-0.106	0.251	0.672	0.899	[0.55, 1.47]	n/a
Education	-0.374	0.363	0.304	0.688	[0.34, 1.40]	n/a
(Constant)	1.340	1.488	0.368	-	-	-

Model χ^2 (8) = 32.2, p < 0.001.
[a] unstandardized regression coefficient

Logistic regression model for PNI.

	**B** [a]	**S.E.**	**p**	**Odds Ratio**	**95% CI for Odds Ratio**	**Reference Category**
Age	-0.033	0.015	**0.033**	0.968	[0.94, 1.00]	n/a
Gender	1.387	0.523	**0.008**	4.004	[1.44, 11.16]	Female
Unilateral/Bilateral	1.750	0.562	**0.002**	5.757	[1.92, 17.30]	Bilateral
Cause of Limb Loss	1.352	0.640	**0.035**	3.866	[1.10, 13.54]	Congenital
Pain Frequency	0.068	0.103	0.511	1.070	[0.87, 1.31]	n/a
Prosthesis Satisfaction	0.110	0.256	0.667	1.117	[0.68, 1.85]	n/a
Functional Satisfaction	-0.497	0.283	0.079	0.609	[0.35, 1.06]	n/a
(Constant)	-0.249	1.312	0.849	-	-	-

Model χ^2 (7) = 44.7, $p < 0.001$.

[a] unstandardized regression coefficient

Logistic regression model for CI.

	**B** [a]	**S.E.**	**p**	**Odds Ratio**	**95% CI for Odds Ratio**	**Reference Category**
Age	-0.050	0.016	**0.001**	0.952	[0.92, 0.98]	n/a
Gender	1.209	0.508	**0.017**	3.350	[1.24, 9.07]	Female
Unilateral/Bilateral	1.323	0.637	**0.038**	3.755	[1.08, 13.08]	Bilateral
Cause of Limb Loss	1.450	0.759	0.056	4.264	[0.96, 18.87]	Congenital
Pain Frequency	0.058	0.097	0.547	1.060	[0.88, 1.28]	n/a
Prosthesis Necessity	0.266	0.177	0.134	1.305	[0.92, 1.85]	n/a
Prosthesis Satisfaction	-0.364	0.203	0.072	0.695	[0.47, 1.03]	n/a
Education	-0.454	0.367	0.216	0.635	[0.31, 1.30]	n/a
(Constant)	-0.428	1.723	0.804	-	-	-

Model χ^2 (8) = 44.7, $p < 0.001$.

[a] unstandardized regression coefficient

Area under ROC curves.

	AUC	p	95% CI
MYO	0.875	< 0.001	[0.758, 0.993]
TMR	0.774	< 0.001	[0.694, 0.854]
PNI	0.826	< 0.001	[0.752, 0.900]
CI	0.811	< 0.001	[0.740, 0.882]
